# Pathways to Identify Electrophiles *In Vivo* Using Hemoglobin Adducts: Hydroxypropanoic Acid Valine Adduct and Its Possible Precursors

**DOI:** 10.1021/acs.chemrestox.2c00208

**Published:** 2022-11-17

**Authors:** Efstathios Vryonidis, Isabella Karlsson, Jenny Aasa, Henrik Carlsson, Hitesh V. Motwani, Marie Pedersen, Johan Eriksson, Margareta Å. Törnqvist

**Affiliations:** † Department of Environmental Science, Stockholm University, SE-106 91 Stockholm, Sweden; ‡ Department of Risk and Benefit Assessment, Swedish Food Agency, SE-751 26 Uppsala, Sweden; § Department of Medical Sciences, Clinical Chemistry, Uppsala University, SE-751 85 Uppsala, Sweden; ∥ Department of Public Health, University of Copenhagen, DK-1353 Copenhagen, Denmark

## Abstract

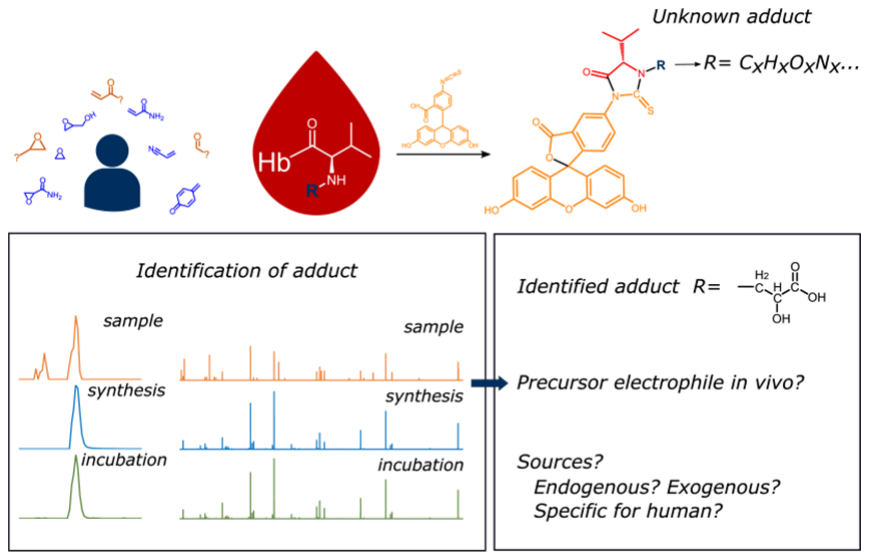

Analytical methods and tools for the characterization of the human exposome by untargeted mass spectrometry approaches are advancing rapidly. Adductomics methods have been developed for untargeted screening of short-lived electrophiles, in the form of adducts to proteins or DNA, *in vivo*. The identification of an adduct and its precursor electrophile in the blood is more complex than that of stable chemicals. The present work aims to illustrate procedures for the identification of an adduct to N-terminal valine in hemoglobin detected with adductomics, and pathways for the tracing of its precursor and possible exposure sources. Identification of the adduct proceeded via preparation and characterization of standards of adduct analytes. Possible precursor(s) and exposure sources were investigated by measurements in blood of adduct formation by precursors *in vitro* and adduct levels *in vivo*. The adduct was identified as hydroxypropanoic acid valine (HPA-Val) by verification with a synthesized reference. The HPA-Val was measured together with other adducts (from acrylamide, glycidamide, glycidol, and acrylic acid) in human blood (*n* = 51, schoolchildren). The HPA-Val levels ranged between 6 and 76 pmol/g hemoglobin. The analysis of reference samples from humans and rodents showed that the HPA-Val adduct was observed in all studied samples. No correlation of the HPA-Val level with the other studied adducts was observed in humans, nor was an increase in tobacco smokers observed. A small increase was observed in rodents exposed to glycidol. The formation of the HPA-Val adduct upon incubation of blood with glycidic acid (an epoxide) was shown. The relatively high adduct levels observed *in vivo* in relation to the measured reactivity of the epoxide, and the fact that the epoxide is not described as naturally occurring, suggest that glycidic acid is not the only precursor of the HPA-Val adduct identified *in vivo*. Another endogenous electrophile is suspected to contribute to the *in vivo* HPA-Val adduct level.

## 1 Introduction

Increasing evidence supports that environmental exposures are major contributors to the development of cancer and other chronic diseases.^1−3^ The importance of environmental factors in cancer etiology was highlighted already in the 1970s by J. Higginson, who considered the environment in a broad view, including lifestyle factors, as diet and cultural habits, as well as chemical exposure.^4,5^ In 2005, the concept of the exposome was proposed by C. Wild and described as the “life-course environmental exposures (including lifestyle factors), from the prenatal period onwards”,^6^ which was later redefined to include exposure from endogenous processes.^7^ The concept was introduced to emphasize the significance of environmental exposures as contributors of common chronic diseases, to balance the focus placed on the genome, and to highlight the need for more holistic methods for exposure assessment.

The exposome concept has stimulated the development and application of advanced analytical methods to characterize environmental chemical exposures in epidemiological studies.^8,9^ In particular, approaches to detect and identify exposures by untargeted analysis with mass spectrometry (MS) of biological samples are rapidly being developed.^10^ The recent decades of MS developments have resulted in improved sensitivity, higher mass resolution, and established software tools and databases that have facilitated the discovery of unknown human exposures to chemicals through MS analysis of biological samples.^11^ Analysis of blood provides the possibility to characterize exposure from external and endogenous sources at concentrations in the blood that could vary at least 10 orders of magnitude between chemicals.^12^

Electrophilic compounds are reactive chemical species characterized by electron deficiency and are of health concern due to their reactivity. Electrophiles react *in vivo* with nucleophilic sites in proteins, DNA, and other biomolecules, and form adducts (reaction products).^13,14^ Analysis of the reactive and short-lived electrophiles *in vivo* is facilitated by analysis of their stable reaction products with biomolecules. Methods to determine adducts to DNA^15^ or blood proteins^16−18^ by MS have been further developed for screening of unknown internal exposure to chemicals, with so-called adductomics approaches. DNA adductomics has been applied in studies of cancer^19−21^ and ecotoxicology.^22^ For the detection of unknown DNA adducts, a method based on the neutral loss of 2′-deoxyribose from digested DNA samples has been used.^22,23^ Recently, algorithms for the detection of such adducts in liquid chromatography–mass spectrometry (LC-MS) analysis have been created.^24,25^ For protein adducts, adductomic approaches have been developed for serum albumin and hemoglobin (Hb).^26^ Rappaport and collaborators have developed a method for the analysis of Cys34 adducts in serum albumin as 21-mer peptides after tryptic digestion.^27^ About 40 modifications to Cys34, such as oxidative modifications and other putative adducts, have been detected with this method. The adduct annotation is aided by software interrogation of tandem mass spectra based on retention time, accurate mass, elemental composition, and using comparison with databases.^28^ This method has been applied in a number of studies including a study on urban ambient air pollution that reported association of adducts with air pollutants, as well as of adducts with health outcome measures.^29^ In another study of occupational exposure to benzene, a positive association was found for putative benzene metabolite adducts.^30^ Furthermore, the Cys34 method has been applied in studies of lung cancer^31^ and childhood leukemia.^32^

**Figure 1:**
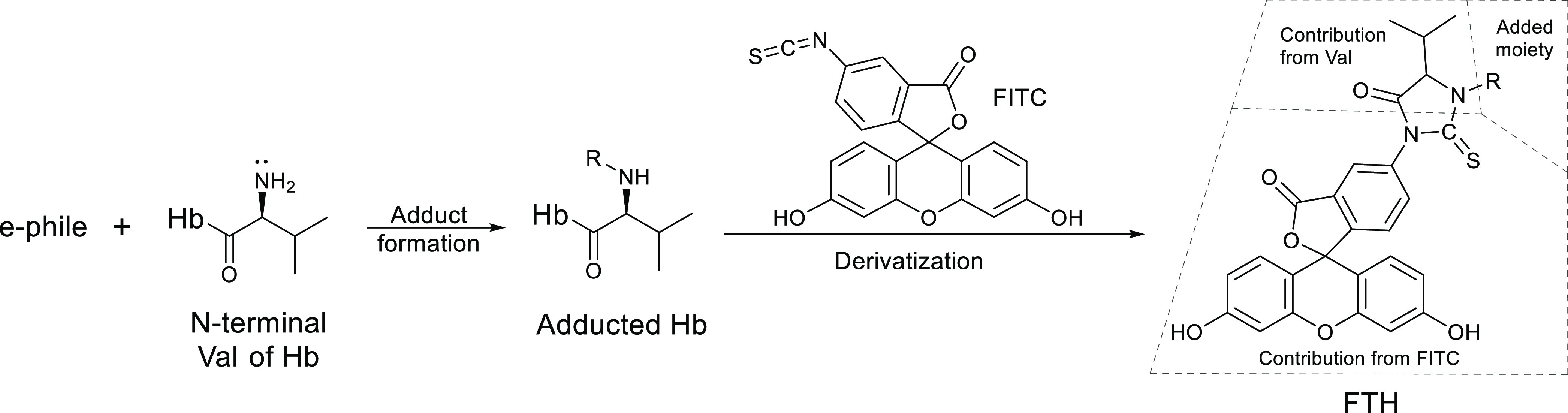
General structure of the fluorescein hydantoin (FTH) derivative formed from the reaction of fluorescein isothiocyanate (FITC) with adducted Hb that detaches the N-terminal valine adduct from the rest of Hb. The adduct is composed of the N-terminal valine from Hb and the added moiety (here represented as the R-group) from the electrophile (e-phile).

Adducts to the N-terminal amino acid valine in Hb can be analyzed after detachment with a modified Edman degradation.^16^ In the present study, the FI*R*E procedure was applied, which is an acronym that stands for the fluorescein isothiocyanate (FITC) reagent that is used for the detachment of the adduct (*R*) as a fluorescein thiohydantoin (FTH) of N-substituted valine with a modified Edman procedure (Figure 1).^33,34^ The FI*R*E procedure has previously been applied by us for targeted measurement of Hb adduct levels from acrylamide (AA), ethylene oxide, etc. in blood from pregnant women and placental umbilical cords.^35^ FTHs of the N-terminal valine adducts exhibit characteristic fragmentation patterns because their structure is partially common (Figure 1). The specific fragmentation pattern was strategically exploited by us in adductomics screening of N-terminal adducts in blood samples from adults.^36^ More than 20 different Hb adducts were detected, of which about half are still unidentified.^37^ The adductomics work for untargeted screening with the FI*R*E procedure has mainly focused on the detection and identification of unknown Hb adducts and their precursor electrophile(s) that are present in humans, and not the association with health effects.^38^

One of the unidentified *in vivo* adducts observed in human blood by LC-MS/MS was the FTH analyte with *m*/*z* 577, which was observed^36^ at somewhat higher levels than the well-studied AA adduct.^39^ In the present work, our aim was to identify this adduct to N-terminal valine in Hb giving the FTH derivative with *m*/*z* 577. We formulated hypotheses on the identity of this adduct and tested these by studying the adduct formation of suggested precursor electrophiles in human blood. For the identification and confirmation of the adduct, we performed analysis with high-resolution mass spectrometry (HRMS) and compared the spectra and chromatogram to those of synthesized and characterized reference substances. Finally, to trace the origin of the precursor electrophile, we determined the Hb adduct levels by HRMS in the blood available from studies of schoolchildren, reference samples from smoking/nonsmoking adults, and exposed rodents. The secondary aim of this work was to explore pathways and demonstrate an approach to reach unequivocal identification of an adduct detected to N-terminal valine in Hb in human blood and for tracing the source of the corresponding *in vivo* precursor electrophile(s).

## 2 Materials and Methods

### 2.1 Chemicals

**Figure 2:**
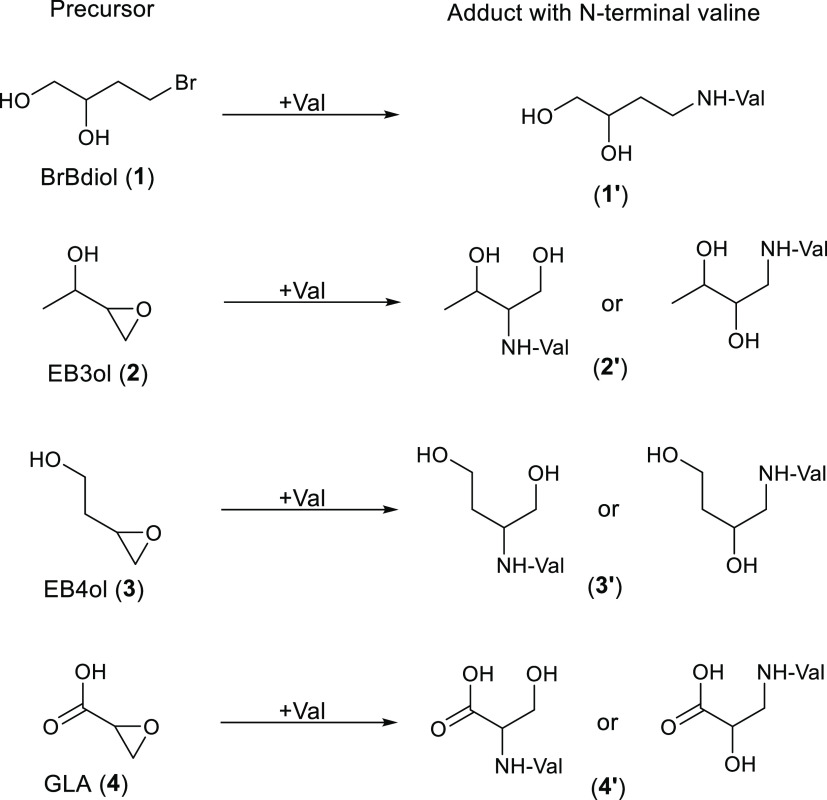
Precursor electrophiles and plausible adducts formed with Val as a model of the N-terminal Val in Hb. Numbers **1**–**4** denote the potential precursors. The prime symbol denotes reaction product with Val without distinction if multiple products are possible. Reaction of precursors **1**–**3** with Val will form five different reaction products that are constitutional isomers (**1′**–**3′**). BrBdiol (**1**) is 4-bromobutane-1,2-diol; EB3ol (**2**) is 2-epoxy-3-butanol; EB4ol (**3**) is 1,2-epoxy-4-butanol; GLA (**4**) is glycidic acid.

4-Bromobutane-1,2-diol (BrBdiol; Figure 2, precursor **1**, CAS: 33835–83–5, 80%); 1,2-epoxy-3-butanol (EB3ol; Figure 2, precursor **2**, IUPAC: 1-(oxiran-2-yl)ethan-1-ol, CAS: 765–44–6, 95%); 1,2-epoxy-4-butanol (EB4ol; Figure 2, precursor **3**, IUPAC: 2-(oxiran-2-yl)ethan-1-ol, CAS: 19098–31–8, 95%); and potassium oxirane-2-carboxylate (potassium salt of glycidic acid, CAS: 51877–54–4, 85%; *cf.* glycidic acid (GLA) in Figure 2, precursor **4**) were purchased from CHEMSPACE (Riga, Latvia). Ethyl 2,3-epoxypropanoate (CAS: 4660–80–4, 98%) was bought from Combi-Blocks (San Diego, CA). Fluorescein isothiocyanate isomer 5 (FITC) was obtained from Karl Industries (Aurora, OH). Valine, *N,N*-dimethylformamide, ammonium hydroxide solution (25%), cyanoacetic acid (99%), formic acid (≥96%), acrylic acid (99%), and l-valine methyl ester hydrochloride (99%) were purchased from Sigma-Aldrich (Steinheim, Germany). Trifluoroacetic acid and sodium hydrogen carbonate were bought from Merck (Darmstadt, Germany). HPLC-grade water, acetone, and acetonitrile were purchased from VWR International S.A.S. (Fontenay sous Bois, France).

### 2.2 Standards

Standard of the FTH of *N*-dihydroxypropylvaline corresponding to the Hb adduct from glycidol (GL-Val-FTH) was synthesized earlier,^40^ and the corresponding isotopically substituted IS, GL-(^13^C_5_)Val-FTH (GL-IS), was synthesized by Aasa et al.^41^ Standards and corresponding isotope-substituted (*d*_7_) internal standards of the FTHs of the valine Hb adducts from AA and glycidamide (GA) were previously synthesized.^34^

### 2.3 Blood Samples

#### 2.3.1 Human Blood Samples

Human blood samples from voluntary schoolchildren, approximately 12 years old, were collected in Sweden in 2014 by the Swedish Food Agency. This study was conducted according to the guidelines of the Declaration of Helsinki. Before donating blood, written informed consent for voluntary participation was given by the participants and by both their parents/legal guardians. The study was approved by the Regional Ethical Review Board, Uppsala, Sweden (Decision Dnr 2013/354; Project Dnr 2372/2013). In the present study, a subset of these samples (as isolated erythrocytes) was used (*n* = 51; males = 35, females = 15, unknown sex = 1). The data collection and biological sampling in this human study are described in earlier studies of this cohort.^42,43^

Blood samples from voluntary healthy adult smokers (*n* = 6) and nonsmokers (*n* = 6) from Sweden were used as reference samples (Ethical approval no. 96–312, Regional Ethical Review Board, Stockholm, Sweden). Commercial whole blood of a nonsmoking adult was obtained from Komponentlaboratoriet, Karolinska University Hospital (Stockholm, Sweden) for use in the *in vitro* studies.

#### 2.3.2 Animal Blood Samples

A few blood samples from earlier animal exposure studies were analyzed to investigate the possible metabolic formation of glycidic acid (GLA) in rodents dosed with glycidol (GL) or AA. GL samples were from three female rats (Sprague Dawley) and three female mice (B6C3F1) from a dosing experiment with GL by gavage, once daily for five consecutive days (0, 37.5, and 75 mg/kg bw/day for the rats and 0, 25 and 50 mg/kg bw/day for the mice).^44^ (Ethical approval license number S7–15, Ethical committee on animal experiments, Swedish Board of Agriculture.) AA samples were from three female Fisher 344 rats from an experiment with AA exposure via drinking water for 7 days (0, 0.1, and 0.5 mg/kg bw/day).^45^ (Approved by the Ethical committee on animal experiments, Stockholm North; no. N/56/02 and N/228/03.)

Bovine blood, used as control sample and for the generation of calibration curves was bought from Håtunalab (Bro, Sweden).

### 2.4 Identification of Unknown Adduct—Generation and Characterization of Reference Adducts

#### 2.4.1 Adduct Formation in Blood *In Vitro* from Potential Precursors 1–3 (Experiment 1)

The adduct formation to N-terminal valine in Hb was studied with the first hypothesized precursor electrophiles, BrBdiol, EB3ol, and EB4ol (Figure 2, precursors **1**–**3**). Stock solutions of the electrophiles were prepared in water and 30 μL were added to hemolyzed erythrocytes from human blood to obtain the final concentrations of 100–1000 μM in 1 mL, and incubated for 1 h (37 °C and 750 rpm). To halt the reaction, the samples were put on ice and were later prepared for adduct analysis (see Section 2.5).

#### 2.4.2 Adduct Formation in Blood *In Vitro* from Potential Precursor 4 (Experiments 2 and 3)

Experiment 2 was performed to confirm whether Hb N-terminal valine in a reaction with GLA (precursor **4**) would form the unknown adduct with *m*/*z* 577 observed *in vivo*. A stock solution was prepared by dissolving 0.8 mg of GLA potassium salt (5.4 μmol GLA) in 155 μL of water (35 mM). An aliquot of 30 or 15 μL, respectively, was added to 1 mL samples of lysed whole human blood. The incubations were carried out for 1 h (37 °C and 750 rpm), and then the samples were put on ice to stop the reaction. The samples were further derivatized and analyzed according to the procedure described in Section 2.5.

Experiment 3 was performed to measure the rate constant for the formation of the adduct of GLA with N-terminal valine in Hb. A single concentration of GLA potassium salt (85 μM GLA) was incubated in lysed whole human blood (1.9 mL, 75 mg Hb/mL; N-terminal valine:GLA, 53:1 molar ratio) at 37 °C and 750 rpm. Samples (250 μL) were collected at different time points (up to 24 h; 7 samples) and put in the freezer to stop the reaction until preparation for adduct measurement (see Section 2.5). The reaction rate constant was estimated from the formed adduct levels.

#### 2.4.3 Synthesis and NMR Characterization of Hydroxypropanoic Acid–Valine–FTH (HPA-Val-FTH)

To confirm the identity of the *in vivo* adduct, a reference compound of **4′** (see Figure 2) was synthesized and characterized. Approximately 200 mg (∼1.7 mmol) of valine and 750 mg of sodium hydrogen carbonate were dissolved in 30 mL of water. The solution was heated to 50 °C and a slight excess amount (∼2 mmol) of ethyl 2,3-epoxypropanoate was added. The progress of the reaction was followed by LC-MS (Instrument 1, Qtrap) until approximately 75% of the valine was consumed. One mL of trifluoroacetic acid was added to hydrolyze the ester bond in the formed adduct and the solution was evaporated to dryness in a rotary evaporator. Acetone (30 mL) was added to the crude product and was left for 1 h under stirring. The sample solution was then filtered, and the acetone was evaporated under a stream of nitrogen. Thereafter, the solid sample was redissolved in 2 mL of acetonitrile/water (1:1) acidified with formic acid. The various substances were then separated on a C18 flash column with acetonitrile/water (1:1) acidified with formic acid as mobile phase, and fractions were collected. The synthetic product expected to contain the hydroxypropanoic acid N-substituted valine (HPA-Val; *cf.*Figure 2, compound **4′**) was evaporated to dryness in a rotary evaporator. The solid product was redissolved in 20 mL of water containing 1.0 g of sodium hydrogen carbonate. The solution was heated to 40 °C and an excess of FITC (∼2 mmol) was added under stirring. The progress of the reaction was followed using the LC-MS (Instrument 1) until approximately 80% of the valine adduct had reacted. The HPA-Val-FTH (*cf.* the general FTH structure in Figure 1) was then isolated by semipreparative LC using a reversed-phase C18-amide column (ACE 5 C18-Amide 100 × 21.2 mm, 5 μm) and isocratic elution (40% methanol in water with 0.2% formic acid) at a flow rate of 7.5 mL/min. The system used was a Shimadzu LC system consisting of two pumps (LC-10 ADvp), an auto-injector (SiL-HTC), and a UV detector (SPD-10 A). Fractions corresponding to the peak of the target compound were collected and further investigated by LC-MS (Instrument 1). The UV detector was set to operate at a wavelength of 274 nm. Thereafter, the solvent was evaporated to dryness in a rotary evaporator at 40 °C. The total yield was 20–30%.

NMR spectra were recorded on a Bruker instrument at 400 MHz (^1^H) and at 100 MHz (^13^C), respectively. Chemical shifts (δ) are reported in ppm, using the residual solvent peak in CD_3_OD (H = 3.31 and C = 49.0) as internal standards, and coupling constants (*J*) are given in Hz.

***HPA-Val-FTH*** (mixture of isomers; see Supporting Information S1 for spectra):

^1^H NMR (400 MHz, CD_3_OD): δ 7.99–7.90 (m, 1H), 7.74–7.63 (m, 1H), 7.36 (dd, *J* = 8.2, 3.7 Hz, 1H), 6.80–6.71 (m, 4H), 6.63 (dd, *J* = 8.7, 2.4 Hz, 2H), 4.80–4.65 (m, 2H), 4.61–4.57 (m, 1H), 3.88–3.83 (m, 0.5H), 3.58–3.49 (m, 0.5H), 2.73–2.55 (m, 1H), 1.37–1.22 (m, 3H), 0.97 (m, 3H).

^13^C NMR (100 MHz, CD_3_OD): δ 184.75, 183.94, 183.18, 173.89, 173.85, 173.77, 169.98, 154.72, 136.80, 136.71, 136.66, 130.68, 129.51, 126.62, 114.40, 111.59, 103.56, 69.61, 69.51, 69.37, 68.97, 30.14, 29.88, 17.86, 17.77, 15.74, 15.71.

#### 2.4.4 Synthesis and NMR Characterization of Additional Reference FTHs

##### 2.4.4.1 H-Val-FTH and AA-Val-FTH

To aid in the NMR characterization of the synthesized reference compound HPA-Val-FTH, two additional reference FTHs, of unsubstituted valine (H-Val-FTH) and of acrylamide-substituted valine (AA-Val-FTH), were synthesized by mixing FITC with Val or AA-Val (systematic name: *N*-(3-amino-3-oxopropyl)-valine), respectively, in the same way as described above.^34^ The NMR analyses of these FTHs are shown in S2 and S3.

##### 2.4.4.2 *N*-(2-Carboxyethyl)–valine–FTH, ACA-Val-FTH

The reference corresponding to the adduct formed from acrylic acid (ACA) with valine was synthesized from L-valine methyl ester hydrochloride (approximately 500 mg, ∼3 mmol) and sodium hydrogen carbonate (800 mg) that were dissolved in 20 mL of water. The solution was heated to 50 °C and a slight excess amount of 220 μL (∼3.2 mmol) of acrylic acid was added. The reaction was followed by LC-MS (Instrument 1, Qtrap) until approximately 75% of the L-valine methyl ester was consumed. The synthesis solution was then evaporated to dryness in a rotavapor, the crude product dissolved in acetone, filtered, and the acetone was evaporated as described in the synthesis description in Section 2.4.3. The solid product was redissolved in 2 mL of acetonitrile/water (1:1) acidified with formic acid. The substances in the product were then separated on a C18 flash column with 35% acetonitrile in water acidified with formic acid as the mobile phase, and fractions were collected. The fractions with the synthetic product were evaporated to dryness in a rotary evaporator. The solid product (60 mg, ∼0.32 mmol) was redissolved in 5 mL of water/isopropanol (1:3) containing 200 mg of sodium hydrogen carbonate. The solution was heated to 40 °C and an excess of FITC (∼0.4 mmol) was added under stirring. The progress of the reaction was followed using LC-MS (Instrument 1) until approximately 80% of the valine adduct had reacted. The ACA-Val-FTH (*cf.* the general FTH structure in Figure 1) was then isolated by semipreparative LC as described in Section 2.4.3. For NMR analysis results, see S4, which confirmed that the product was ACA-Val-FTH, but also contained H-Val-FTH. As this reference was used to confirm the retention time of the ACA-Val-FTH, the purity was determined to be sufficient.

### 2.5 Derivatization of Blood Samples Using the FI*R*E Procedure and Quantification of Adduct Levels

#### 2.5.1 General Procedures

Blood samples were prepared for analysis according to the FI*R*E procedure^46^ involving derivatization overnight with FITC for detachment of N-terminal valine adducts.^34^ Before derivatization of the samples, the Hb concentration was measured with HemoCue Hb 201+ system. Sample volumes of 250 μL blood were derivatized with 5 mg of FITC overnight, followed by protein precipitation with acetonitrile before clean-up of the supernatant with SPE (Oasis MAX, Waters MA). The eluates of the samples were evaporated to dryness and reconstituted in 40% acetonitrile before analysis by LC-MS. The internal standard, GL-(^13^C_5_)Val-FTH (GL-IS), was added before the SPE clean-up. The calibration curve (CC) used for quantitation of the adduct levels from GLA, GL, AA, GA, and ACA, was made by adding four levels of GL-Val-FTH STD and GL-IS in bovine blood. The CC samples, without derivatization, were directly cleaned up by SPE according to the FI*R*E procedure. Duplicate method blank samples were made from bovine blood, with only IS added. Quantification of the adduct levels in the samples was made by normalizing the response of the analytes to the GL-IS response and the GL CC.

#### 2.5.2 Blood Samples from *In Vitro* Experiments

The samples from Experiments 1–3 were derivatized using the FI*R*E procedure according to the above. For the determination of formed adducts by LC-MS analysis, Instrument 2 (Orbitrap) was used for Experiments 1–2 and Instrument 1 (Qtrap) for Experiment 3.

#### 2.5.3 Blood Samples from Human Volunteers

The blood samples from schoolchildren (*n* = 51) and adult smokers/nonsmokers (*n* = 6/6), as well as control bovine blood, were processed according to the FI*R*E procedure described above. The samples were analyzed by LC-MS (Instrument 2) and quantified as described above.

#### 2.5.4 Blood Samples from Exposed Animals

The samples (*n* = 3 per study) were derivatized as described above with the following deviations: The volume of blood used for derivatization with FITC was 150 μL, and the amount of FITC used per sample was 3 mg. The samples were analyzed and quantified on LC-MS Instrument 2.

### 2.6 Analysis of FTH Compounds by LC-MS

#### 2.6.1 Instrument 1, Qtrap

The system was composed of a Shimadzu Prominence LC20 system (Shimadzu Corp., Kyoto, Japan) coupled to a 3200 Qtrap mass spectrometer (AB Sciex, Ontario, Canada). The column used was a Supelco Discovery HS C18, 2.1 × 150 mm, 3 μm, and the mobile phases were A: 10% acetonitrile in water with 0.1% formic acid and B: 100% acetonitrile with 0.1% formic acid. The gradient elution program was at 0.15 mL/min and started at 20% B, followed by a ramp to 100% B in 18 min and kept for 7.5 min, then reduced to 20% B in 0.5 min and kept at 20% B for 4 min. The MS was operated in ESI^+^ using multiple reaction monitoring (MRM). The collision energy was set to 50 V. Curtain gas was set to 40 and the ionization spray at +5000 V. The source temperature was set to 450 and gases 1 and 2 were set to 20 and 15, respectively. Declustering potential was set to 95.

#### 2.6.2 Instrument 2, Orbitrap

The LC-MS/HRMS system was consisting of a Dionex Ultimate 3000 UHPLC system connected to a Q-Exactive HF Quadrupole-Orbitrap hybrid MS System (Thermo Fisher Scientific, MA). The column used for the analysis was an Acquity UPLC HSS C18 (2.1 × 100 mm, 1.8 μm). Mobile phase A composed of 10% acetonitrile and 0.1% formic acid in water and mobile phase B composed of 10% water and 0.1% formic acid in acetonitrile. Gradient elution was used with 0.3 mL constant flow and the program starting at 30% B for 0.5 min, then 100% B in 7 min, and kept at 100% B for 2 min, followed by decrease to 30% B in 0.5 min and kept for 2 min for column reequilibration before the next run. Parallel reaction monitoring mode was used for this analysis with the resolution set to 60,000 and operated in ESI mode at +4000 V. The normalized collision energy was set to 45 and the capillary temperature was set to 275 °C. Maximum injection time was set to 100 ms and auto gain control to × 10^5^. The S-Lens RF Level was set to 60. The sheath gas was set to 20, and the auxiliary gas was set to 10.

## 3 Results and Discussion

### 3.1 Identification of the Unknown Adduct with *m*/*z* 577

#### 3.1.1 Comparison of *In Vivo* Unknown Adduct with Adducts from Suspect Precursors

The investigation to identify the adduct with *m*/*z* 577 detected in the previous adductomics screening with the FI*R*E method started with suggesting precursor electrophiles based on unit resolution MS data. The first experiment concerned BrBdiol, EB3ol, and EB4ol (Figure 2, precursors **1**–**3**). The theoretical log *P* of the corresponding R-Val-FTHs was in the range of the expected log *P* of the *in vivo* observed adduct (data not shown). The ability of these electrophiles to form stable adducts to N-terminal valine of Hb in blood (see Figure 1) with chemical formula fragment C_4_H_9_O_2_^•^ (see also adducts **1′–3′** in Figure 2) was studied and verified by MS. The retention times (Rt) of these C_4_H_9_O_2_-Val-FTHs (five constitutional isomers in total) showed that none of the adducts matched the Rt of the adduct observed *in vivo*. This was corroborated with later HRMS analysis showing that the FTH analytes of the adducts **1′–3′** had *m*/*z* of 577.16391 (C_4_H_9_O_2_-Val-FTH), and did not match the observed unknown *in vivo* adduct, which *m*/*z* corresponded to C_3_H_5_O_3_-Val-FTH.

From the information of the HRAM spectrum, it was proposed that the unknown adduct could correspond to the hydroxypropanoic acid–valine (HPA-Val) adduct formed from glycidic acid (GLA) as shown in Figure 2 (precursor **4**). The adduct generated by incubation of blood with GLA (Experiment 2) was in accordance with the *in vivo* observed adduct with regards to Rt and accurate mass (theoretical *m*/*z* = 577.12753). For confirmation of the identity, the FTH of HPA-Val adduct (mixture of isomers, **4′**) was synthesized and characterized with NMR (see also Section 3.1.2 and S1).

**Figure 3:**
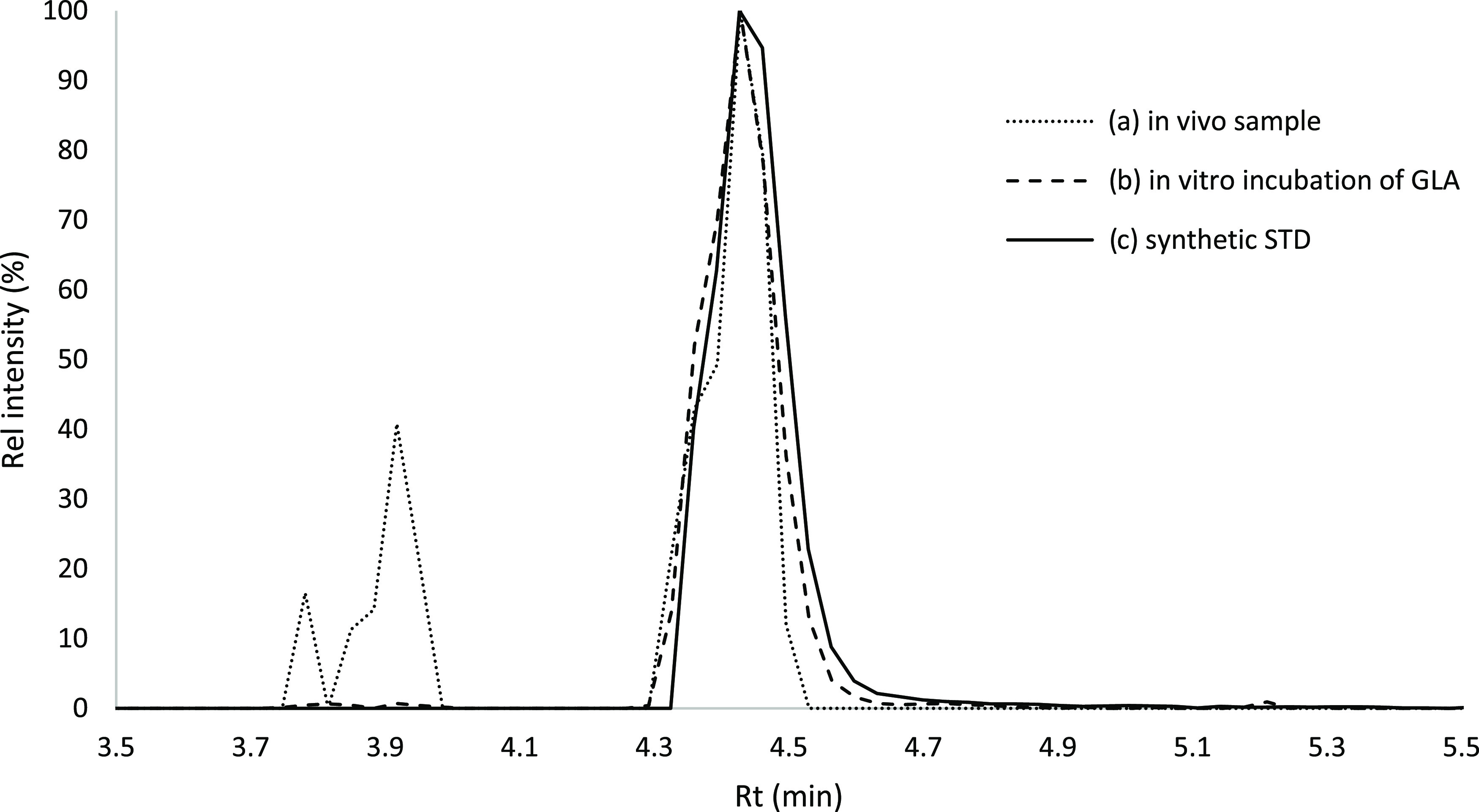
Superimposed HRMS chromatograms for the analyte HPA-Val-FTH from three different runs: (a) *in vivo* sample, (b) *in vitro* sample from the incubation in blood with GLA, and (c) synthetic STD; (normalized to 100% between the runs).

**Figure 4:**
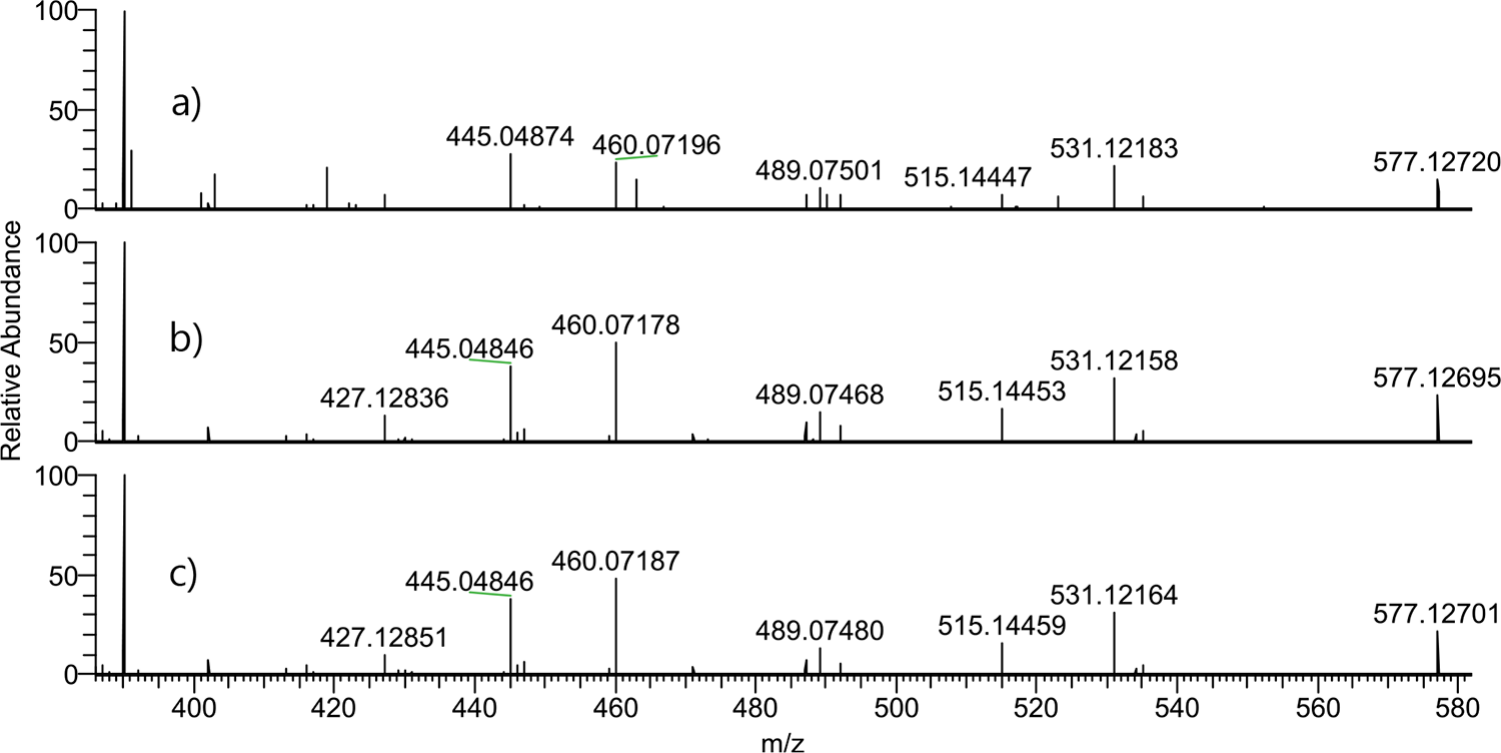
Extracted HRAM MS2 spectra for the peak at 4.43 min in the 577 PRM experiment for: (a) *in vivo* blood sample, (b) *in vitro* sample from incubation in blood with GLA, and (c) synthetic STD.

In Figure 3, superimposed chromatograms of the unknown adduct observed *in vivo*, the adduct from the *in vitro* incubation of GLA in blood, and the synthesized standard of HPA-Val-FTH are shown for *m*/*z* = 577.12753 (*m*/*z* range = 3 ppm). It was observed that the Rt of the respective peak in these three samples are matching. Also, as observed in the HRAM experimental spectra in Figure 4, the fragmentation pattern matches for all three samples. Some minor deviations are observed in the fragmentation pattern in the *in vivo* sample, which could be explained by the much lower analyte level in relation to the biological matrix.

**Figure 5:**
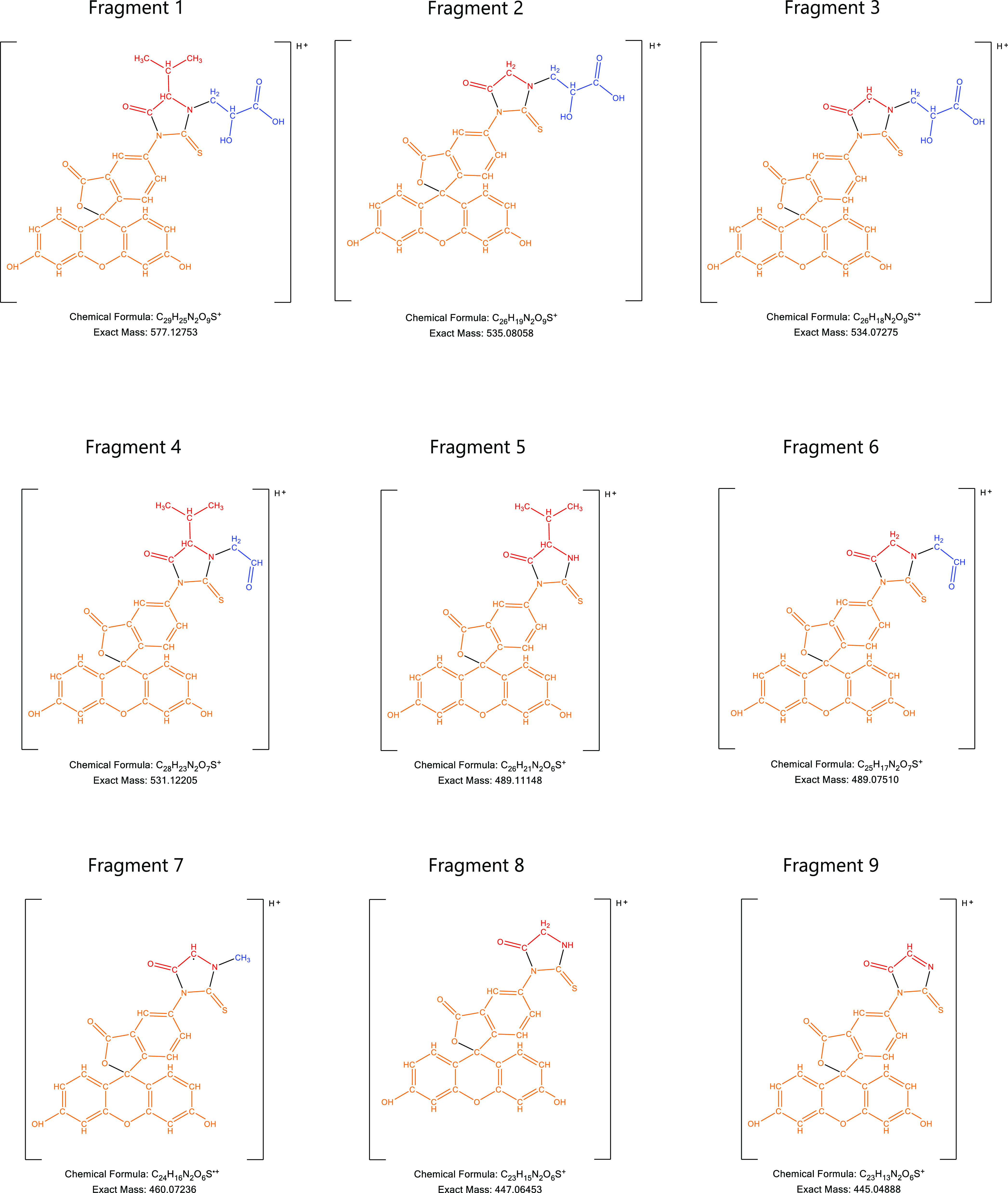
Suggested fragment structures for the major observed fragments for the *in vivo* sample, the *in vitro* incubation in blood with GLA sample, and synthetic STD.

Table 1 lists the observed accurate mass measurements from the HRMS analyses of the studied adduct in an *in vivo* sample, the adduct from *in vitro* incubation with GLA in blood, and the synthesized standard. The chemical formulas identified and the theoretical *m*/*z* values are listed in comparison. Figure 5 shows the suggested fragment structures.

**Table 1:** Observed Molecular and Fragment Ions in Comparison to Theoretical *m*/*z* and Corresponding Chemical Formulas for the *In Vivo* Sample, the *In Vitro* Incubation in Blood with GLA Sample, and Synthetic STD

theoretical *m*/*z*	observed *m*/*z*			identified chemical formula	ID	difference in ppm to theoretical *m*/*z*		
	*in vivo* sample	*in vitro* incubation sample	synthesized standard			*in vivo* sample	*in vitro* incubation sample	synthesized standard
577.12753	577.127(20)	577.126(95)	577.127(01)	C_29_H_25_N_2_O9_S_^+^	fragment 1^a^	–0.6	–1.0	–0.9
535.08058	535.079(47)	535.080(02)	535.080(14)	C_26_H_19_N_2_O_9_S^+^	fragment 2^a^	–2.1	–1.0	–0.8
534.07275	NOT FOUND	534.072(20)	534.072(63)	C_26_H_18_N_2_O_9_S^.+^	fragment 3^a^^,^^b^	N/A	–1.0	–0.2
531.12205	531.121(83)	531.121(58)	531.121(64)	C_28_H_23_N_2_O_7_S^+^	fragment 4^a^	–0.4	–0.9	–0.8
515.14489	515.144(47)	515.144(53)	515.144(59)	C_28_H_23_N_2_O_8_^+^	unidentified fragment	–0.8	–0.7	–0.6
489.11148	489.111(45)	489.109(99)	489.110(26)	C_26_H_21_N_2_O_6_S^+^	fragment 5^a^^,^^b^	–0.1	–3.0	–2.5
489.07510	489.075(01)	489.074(68)	489.074(80)	C_25_H_17_N_2_O_7_S^+^	fragment 6^a^	–0.2	–0.9	–0.6
460.07236	460.071(96)	460.071(78)	460.071(87)	C_24_H_16_N_2_O_6_S^.+^	fragment 7^a^^,^^b^	–0.9	–1.3	–1.1
447.06453	447.061(71)	447.064(09)	447.064(15)	C_23_H_15_N_2_O_6_S^+^	fragment 8^a^	–6.3	–1.0	–0.8
445.04888	445.048(74)	445.048(46)	445.048(46)	C_23_H_13_N_2_O_6_S^+^	fragment 9^a^^,^^b^	–0.3	–0.9	–0.9
390.04307	390.042(66)	390.042(54)	390.042(63)	C_21_H_12_NO_5_S^+^	reagent	–1.1	–1.4	–1.1

a See Figure 5 for the suggested structure.

b Expected from the literature.^36^

As here exemplified, HRMS enables the deduction of the chemical formulas of unknown adducts and aids in confirming the identity of the adduct. However, only HRMS is not enough for unequivocal identification, reference compounds are also necessary, a fact that has been emphasized by, *e.g.*, Sabbioni et al.^47^ This was illustrated in the current work by the different adducts **1′–3′** (Figure 2) that all give adduct analytes with the same exact mass. For unequivocal identification of an unknown adduct analyte, the comparison by MS with a synthesized and characterized standard is the final step, as performed in this work.

#### 3.1.2 Synthesis and Characterization of Reference Compounds

The identity of the synthesized reference of the unknown adduct as HPA-Val-FTH was characterized by NMR (^1^H NMR and ^13^C NMR). In addition to HPA-Val-FTH, the FTHs of unmodified Val (H-Val-FTH) and of acrylamide-substituted Val (AA-Val-FTH), were synthesized and characterized by NMR to be used as references for the interpretation of the more complex NMR spectra of HPA-Val-FTH. In addition, ACA-Val-FTH was synthesized to be used as reference for analysis of the corresponding adduct in blood from schoolchildren. The NMR spectra for the synthesized FTHs are shown in S1–S4. The suggested interpretation of HPA-Val-FTH from comparison with the reference FTHs (see S5), verified the identity of the new product as HPA-Val-FTH. The NMR spectra for the reference compound H-Val-FTH were in accordance with the literature.^33^

The synthesis of reference compounds of FTH derivatives of N-substituted valines is a two-step reaction process where the product of the first reaction is the valine adduct, which is then reacted with FITC to form the desired FTH. In the present work, nucleophilic substitution by an epoxide was the pathway chosen to synthesize *N*-hydroxypropanoic acid–valine, HPA-Val. The ester derivative of GLA (S6a) was preferred over GLA (precursor **4** in Figure 2) to avoid the reaction of the carboxylic acid group of GLA with the amino group of valine, and thus maximize the yield of the desired S_N_2 product. Initially, alkylation by alkyl halide was tested for the synthesis of HPA-Val, using the reaction of isoserine with 2-bromoisovaleric acid (see S6b), which is a synthetic pathway that has been used for N-substituted valines before.^48,49^ However, the yield of the product in this case was deemed too low (data not shown). Reductive amination via Schiff base formation to yield HPA-Val was not explored (see S6c) as the relevant aldehyde was not commercially available. That pathway has previously been applied for the synthesis of N-substituted valines with satisfactory yield (*e.g.*, for the synthesis of GL-Val used as IS in the present work).^41^

### 3.2 Occurrence and Origin of the Hydroxypropanoic Acid Val in Hb *In Vivo*

#### 3.2.1 Outline of Studies

Characterization of exposure to electrophilic compounds through identification of adducts in blood samples could be complex. Even though the *in vitro* experiments with an electrophile may give confident results regarding the identity of the observed *in vivo* adduct, the actual *in vivo* precursor electrophile may not be the same. In addition, there could be multiple precursor electrophiles that form the same adduct. An electrophile, or a compound which biotransforms to an electrophile, could either originate from external exposure or from endogenous processes like oxidative stress. Some exposure sources, as tobacco smoking, are common for multiple adduct-forming electrophiles.

**Figure 6:**
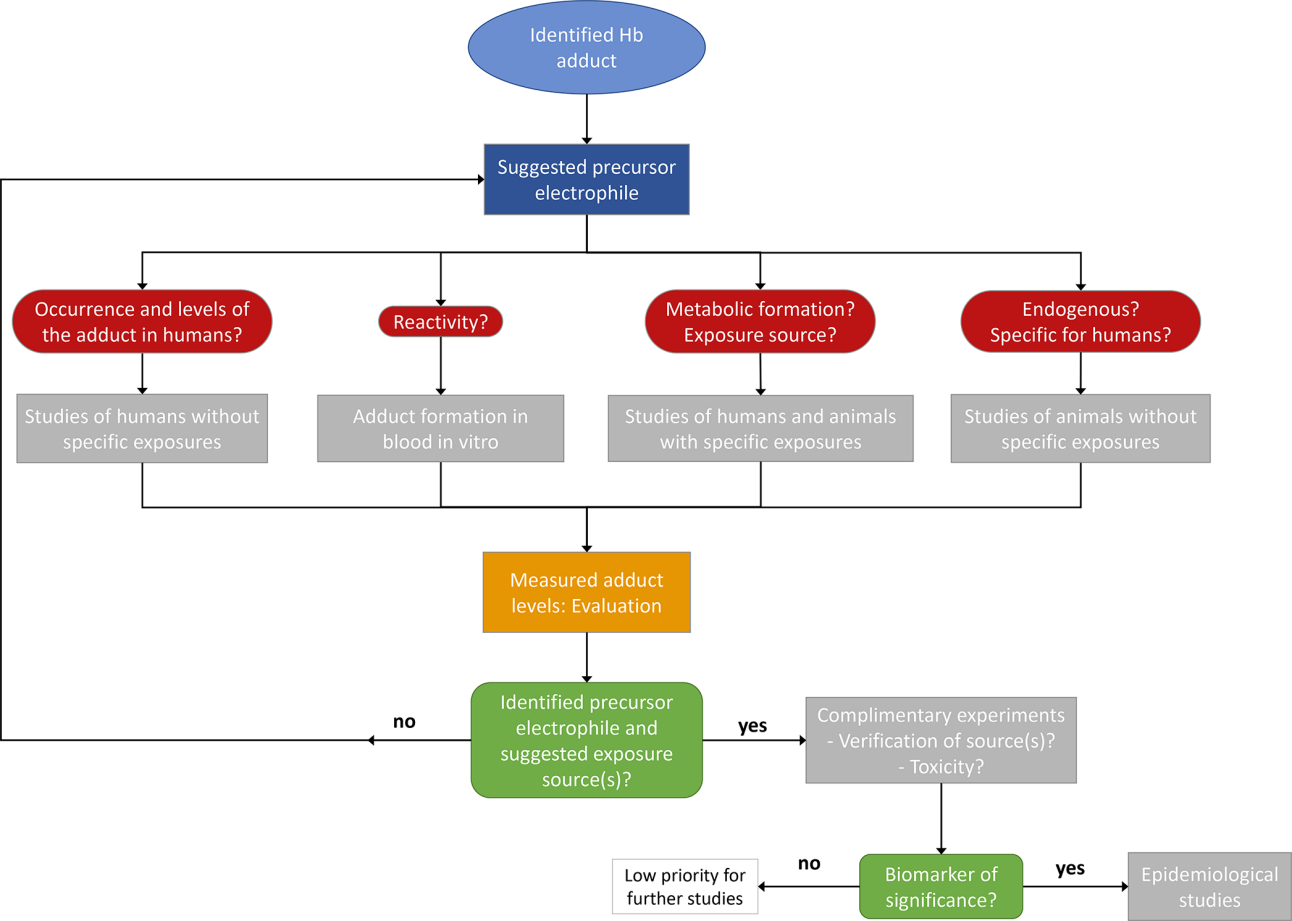
Suggested strategy for the identification of precursors and exposure sources of an identified Hb adduct. Typical questions asked and type of studies to be performed to answer these questions are indicated. Measurement of adduct levels in in vitro and *in vivo* samples is the primary method in the first steps of the investigation. When a precursor electrophile is identified and exposure source of the observed *in vivo* adduct can be suggested, complimentary experiments of the electrophile/parent compound (occurrence in exposure and assessment of toxicity) should follow to determine whether the biomarker/adduct is of significance for application in epidemiological studies.

In the present work, we studied a few possible parent compounds to the suggested electrophilic precursor GLA and hypothetical exposure sources to the identified HPA-Val adduct, as outlined in Figure 6. The level of this adduct was compared in reference samples from humans and animals, with or without specific exposure, as well as in samples from *in vitro* studies, for elucidating the origin of the identified adduct. Follow-up experiments should include complimentary verification investigations to evaluate if the adduct is of significance as a biomarker of exposure or of toxicity, which should be monitored in future epidemiological studies.

#### 3.2.2 Occurrence of the Hydroxypropanoic Acid Adduct to N-Terminal Valine in Hb in Schoolchildren Blood Samples

**Figure 7:**
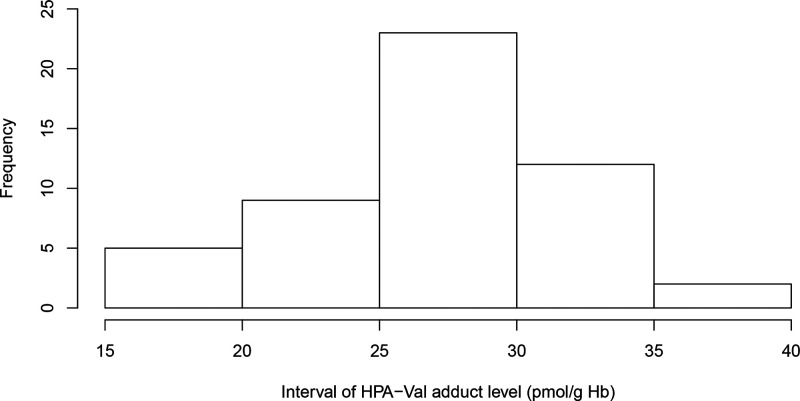
Distribution of the adduct levels in schoolchildren (*n* = 51; analysis for one sample under LOQ) for HPA-Val-FTH.

The FTH of the HPA valine adduct (HPA-Val-FTH) with *m*/*z* of 577.12753 was observed in all blood samples from schoolchildren in the analysis. The adduct level distribution ranges between 16 and 38 pmol/g Hb as shown in Figure 7. This is a semiquantitative measurement because the used IS and CC was of another FTH, namely, GL-Val-FTH. The repeatability of the method, as relative standard deviation, was estimated to be 16–32% in quintuple samples from three of the individuals. Before application, *e.g.*, in epidemiological studies, the analytical method for HPA-Val-FTH should be properly evaluated with a dedicated IS and CC (as performed for the analysis of GL adducts in this sample material with the same method).^41^

#### 3.2.3 Estimation of the Rate Constant for Adduct Formation by Glycidic Acid

To elucidate if GLA, with regard to its reactivity, could be a precursor of the HPA-Val (precursor **4** and adduct **4′** in Figure 2) observed *in vivo*, the rate constant of adduct formation toward N-terminal valines in Hb was estimated. This was studied *in vitro* under pseudo-first-order reaction conditions in whole lysed blood with GLA (Experiment 3). The experiment was followed for 24 h, to evaluate whether secondary reactions of the formed adduct occur and to get an estimation of the stability of GLA in blood.

The plot of the normalized response levels of the HPA-Val-FTH vs time of incubation showed coefficient of determination >0.99 (see S7), which did not appear to plateau. Thus, the concentration of GLA appeared relatively constant during the 24 h experiment, which indicated that there was no observable hydrolysis or other degradation reaction of the epoxide under these conditions. The reaction rate (*k*_Val-GLA_) obtained during these experimental conditions was estimated to be approximately 0.5 pmol/g Hb per μM × h. This would indicate a reaction rate constant that is about 10 times lower than the *k*_Val_ measured for AA^50^ and about 40 times lower than the reaction rate for the epoxide GL.^41^ According to these results GLA could not be excluded as a precursor contributing to the identified HPA-Val adduct *in vivo*.

#### 3.2.4 Possible Formation of Glycidic Acid as a Metabolite: Comparison of Adduct Levels *In Vivo*

**Figure 8:**
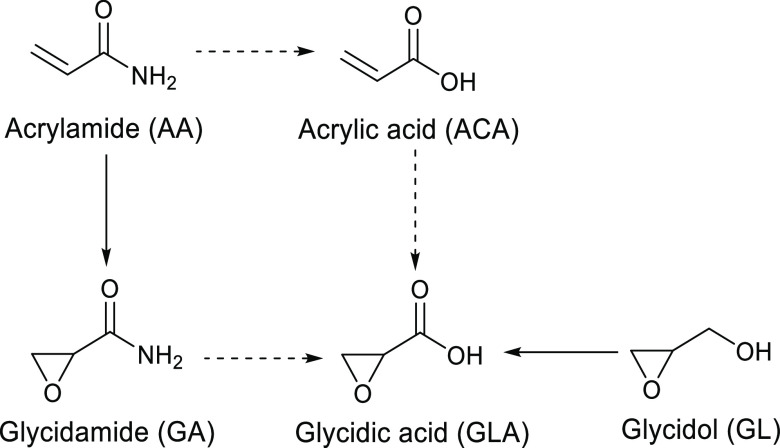
Proposed possible formation routes of GLA by ACA, GA, and GL *in vivo*. Solid arrows denote observed metabolic reactions, and dashed arrows denote the proposed not earlier observed formation routes.

In the literature, no information supporting the occurrence of GLA in humans or exposure to humans was found. Therefore, we continued to investigate whether the adduct had any relation to other 3-carbon electrophiles present as adducts from some other common exposures in humans. The three-carbon electrophiles shown in Figure 8, AA and its metabolite GA, GL, and ACA, are observed regularly as adducts to N-terminal valine in Hb in blood from nonsmokers. The levels of the three first adducts are increased in smokers (AA;^51,52^ AA/GA;^39^ GL;^41,53^ ACA^38^). AA, GL, and ACA are shown to be present in different foods (GL;^54,55^ AA;^56−58^ ACA^59^). AA and GL are also components of tobacco smoke.^60−62^ GL and AA are classified as probable carcinogens to humans (group 2A) by the International Agency for Research on Cancer, and ACA has been considered not classifiable as to its carcinogenicity to humans (group 3).^63^ These electrophiles could possibly be metabolized to GLA. However, in the literature, evidence was only found for the metabolism of GL to GLA in bacteria.^64^ Therefore, levels of the HPA-Val adduct (which would be formed from GLA), and adduct levels to N-terminal valine from the other electrophiles in Figure 8, were compared in blood samples from humans and rodents, in an effort to trace the origin of the HPA-Val adduct.

**Figure 9:**
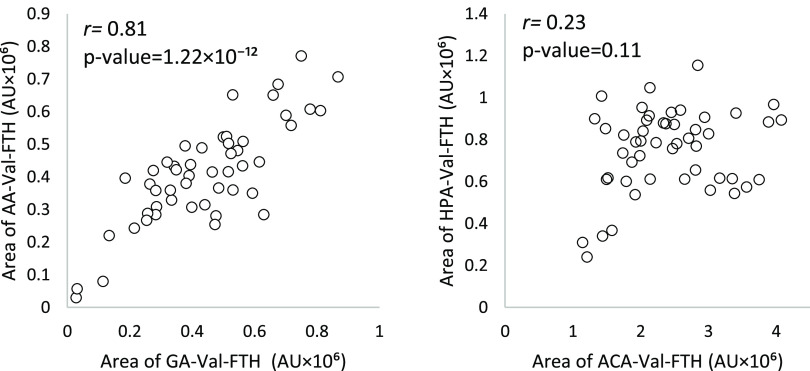
Correlation plots (Pearson’s r) using the area of the FTH analytes obtained from LC-MS analysis for (left) AA-Val against GA-Val and (right) HPA-Val against ACA-Val, in the schoolchildren samples (*n* = 50).

The peak areas from the LC-MS analysis of the adduct analytes from the different electrophiles (AA, GA, ACA, GL, and GLA) in the samples from schoolchildren were compared. The HPA-Val-FTH levels showed a weak positive correlation (*r* = 0.23) only with the levels of the ACA-Val-FTH levels (Figure 9, right), but there was no significant linear relationship (*p*-value of 0.11, with α = 0.05 and *n* = 50). As a comparison, for the same samples, the levels of AA-Val-FTH and GA-Val-FTH showed a strong positive correlation (*r* = 0.81, Figure 9, left), with a *p*-value of 1.22 × 10^–^^12^. A strong correlation between AA and its metabolite GA was expected as it has been observed earlier, despite individual variation in the ratio of adduct levels,^65^ primarily due to polymorphism of the metabolic enzyme CYP2E1.^66^ The comparison of adduct levels from the electrophiles in Figure 7 in the blood samples from schoolchildren thus gave no clear indication concerning the origin of the HPA-Val adduct.

**Figure 10:**
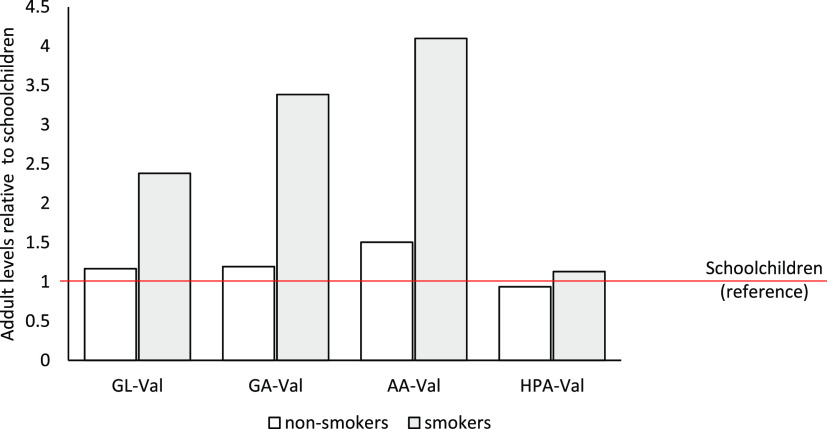
Val adduct levels corresponding to glycidol (GL), glycidamide (GA), acrylamide (AA), and glycidic acid (GLA) in reference blood samples from adults (6 smokers, 6 nonsmokers) normalized to the mean adduct levels in samples from schoolchildren (*n* = 51).

In the next step, we compared the adduct levels in blood samples from the group of schoolchildren with adduct levels in a few blood samples from adult smokers and nonsmokers (previously used as reference samples).^41,67^ This was to investigate if the HPA-Val adduct level follows the same trend as AA, GA, and GL adduct levels that were statistically different between nonsmokers and smokers. In Figure 10, the mean levels of these adducts in the samples of adult smokers (*n* = 6) and nonsmokers (*n* = 6) are presented normalized to the mean of the schoolchildren samples (*n* = 50). One sample t-tests revealed that for the level of the FTH of HPA-Val, no significant difference between schoolchildren and the respective groups of adult smokers and nonsmokers was observed (*p*-value = 0.53 and 0.52, respectively; α = 0.05). In contrast, the mean levels of the adducts from GL, AA, and GA are much higher in smokers compared to the two other groups (Figure 10). The lack of increase of HPA-Val level in smokers, suggests that GLA or any other HPA-Val adduct precursors are not present in tobacco smoke at any significant level.

To investigate further the hypothesis whether GLA could be a metabolite of GA (from AA) or GL, a few blood samples from rodents exposed to AA or GL at high doses were analyzed for the HPA-Val adduct. HPA-Val was observed in all of the blood samples, including the controls. No increase of the HPA-Val adduct was detected in AA-exposed rats (adduct of levels from AA and GA up to 0.7 and 1.7 nmol/g Hb, respectively).^45^ In contrast, the samples from all of the GL-exposed mice and rats (*n* = 4), showed a small but clear increase of the HPA-Val adduct levels (the increase was in the order of 0.1% of the respective GL adduct level) (see S8). (The adduct levels from GL were in the range of 4–26 nmol/g Hb^44^). These results suggest that GLA is not a metabolite of AA/GA and that it is a minor metabolite of GL. Further, the HPA adduct was present in the samples from all (*n* = 3) controls of mice and rats.

#### 3.2.5 Summary of Findings Regarding Tracing of the Adduct Precursor and Its Origin

In summary, based on the human exposures to the three-carbon electrophiles in Figure 7, their contribution via biotransformation to the HPA adduct levels is judged to be none or negligible. The precursor to the HPA adduct does not appear to have the same exposure sources, *e.g.*, tobacco smoke, as these electrophiles. The estimated rate constant for the formation of the Val adduct is much lower for GLA than for AA, although the HPA-Val and AA-Val adducts are observed at comparable levels in humans. This indicates that if GLA is the only source of the observed HPA-Val adduct, its concentration over time in humans would be higher than that of AA, which seems less likely. Moreover, the HPA-Val Hb adduct is observed in both humans and animals without specific exposure, suggesting that the adduct originates from endogenous precursor(s), of which the studied GLA could be one of the contributors.

The plausibility for the formation of stable protein adducts from electrophiles *in vivo* depends on the occurrence of the precursor and if the *in vivo* conditions enable the adduct formation. 2-Hydroxy-3-oxopropanoic, also named tartronate semialdehyde, occurs *in vivo* and has been detected in several different foods.^68^ If the Schiff base intermediate, expected to be formed from tartronate semialdehyde with N-terminal valine in Hb is then reduced to the HPA-Val adduct, this aldehyde could also be a precursor (see S6c). We have earlier shown that the reduction of a Schiff base formed to N-terminal valine from 4-hydroxybenzaldehyde, could proceed directly in blood *in vitro* under physiological conditions.^69^ To determine whether tartronate semialdehyde could be a source of HPA-Val *in vivo*, comprehensive complementary *in vitro* and *in vivo* studies would be required as indicated in Figure 6.

#### 3.2.6 Significance of Identified Adduct and Corresponding Exposure to Precursor Electrophiles

The potential toxicological significance of an identified adduct depends on the reactivity of the precursor, which differs substantially between an epoxide and an aldehyde, discussed above as precursors of the HPA-Val adduct. The current results suggest that the HPA adduct is a reaction product of normal metabolism, and not related to external exposure. Furthermore, the levels of HPA-Val in the studied group of children are not indicated to deviate from groups of smoking and nonsmoking adults. The HPA-Val adduct should be further studied; even though this adduct does not appear to be of high priority for human monitoring, considering the large number of adducts that are still unidentified.

It is important that different types of control samples are used in adductomics screening, as was done when this adduct was first detected.^36^ This is because some adducts from low-molecular-weight electrophiles could be artificially formed during the processing or storage of biological samples.^70^ This could be a pitfall when analyzing Hb adducts at low levels (1–10 per 10^7^ N-terminal valines) in humans. There has been no indication that the HPA-Val adduct is formed *in vitro* during the processing of samples, and furthermore, an advantage of using modified Edman procedures for analysis is that N-substituted valine in Hb cannot be misincorporated during protein synthesis.^71^

### 3.3 Applicability of the Approach for the Identification of Exposure to Electrophiles

Sensitivity is the main advantage of the modified Edman-based methods for the characterization of exposure to electrophiles. Although the methodology has been applied to many low-molecular electrophiles, it does not work for all electrophiles. One limitation is that certain N-terminal valine adducts cannot be detached from Hb with Edman reagents, *e.g.*, the adduct from diepoxybutane,^72^ which has been analyzed as a modified N-terminal peptide.^73^ Bulky electrophiles, such as those of polycyclic aromatic hydrocarbons, react with other nucleophilic sites, *e.g.*, His in serum albumin.^74,75^ Other examples are arylamines and nitroarenes, monitored as specific Cys adducts in Hb.^76^ Cys34 in serum albumin has been shown to be useful in monitoring oxidative stress-induced adducts.^30^ The variation in the reaction pattern of electrophiles toward nucleophilic sites in proteins emphasizes that further analytical development is needed for the detection/identification of adducts in blood proteins, to expand the knowledge of the exposome and trace exposure to electrophiles that could be important for health effects. For instance, in addition to Val adducts, Cys and His adducts are formed in Hb from simple alkylating agents, like ethylene oxide, which was demonstrated early with radioactive labeling.^77^ Adduct formation of aldehydes is less characterized, but Schiff base formation to amino groups, which could give stable adducts through secondary reactions, could be expected to be major adducts formed in proteins. Proteomics approaches are now used to profile electrophile reactivity based on the adduct formation pattern in Hb and serum albumin.^78−80^ One example is 1,2-epoxy-3-phenoxypropan, which in a proteomics study shows only Val and His adducts in Hb and Cys adducts in serum albumin.^79^ Regarding aldehydes, benzaldehyde is shown to form adducts in Hb to certain Tyr, Thr and Ser, in addition to His and Val, after reduction,^78^ and recently Lys525 in serum albumin was suggested as a monitor site for aldehydes.^81^ Proteomics approaches to characterize the exposome are initiated^81,82^ but the sensitivity needs further improvement for application in population studies for the detection of adducts formed by electrophiles from external sources.

This study illustrates the complexity of the identification of a protein adduct *in vivo* and its precursor electrophile and origin. Verification with a synthesized standard was used as unequivocal proof of the adduct identity. This contrasts with typical exposomics studies that use high-throughput methods to detect chemicals in biological samples, which generate data for a large number of unknowns, and rely on databases and processing software for the identification of compounds. Detection of putatively identified adducts by adductomics screening in epidemiological studies and study of their association to exposure sources or markers of health is an alternative to the approach based on multianalyte targeted screening of identified adducts (Figure 6). Considering the large number of potential hazardous chemicals in our external environment as well as endogenous compounds, high-throughput methods using putative identification are necessary to capture the broader picture of the exposome. The unequivocal identification of detected compounds is necessary when a chemical observed as an adduct *in vivo* is suspected to have a potential of toxicity or other significance.

## Supporting Information Available

NMR spectra of the synthesized HPA-Val-FTH (S1:); NMR analysis of the synthesized reference of H-Val-FTH (S2); NMR analysis of the synthesized reference of AA-Val-FTH (S3); NMR analysis of the synthesized reference ACA-Val-FTH (S4); comparison of the NMR spectra of HPA-Val-FTH and the reference FTHs (S5); illustration of synthetic pathways to HPA-Val (S6); experiment in blood to estimate the rate constant for HPA-Val adduct formation from GLA (S7); and HPA-Val adduct levels in rodents dosed with glycidol or acrylamide (S8) (PDF)

## Abbreviations

AA: acrylamide
ACA: acrylic acid
bw: body weight
CC: calibration curve
FITC: fluorescein isothiocyanate
FTH: fluorescein thiohydantoin
GA: glycidamide
GL: glycidol
GLA: glycidic acid
Hb: hemoglobin
HRAM: high-resolution accurate mass
HPA: hydroxypropanoic acid
IS: internal standard
LC: liquid chromatography
MRM: multiple reaction monitoring
(HR)MS: (high-resolution) mass spectrometry
NMR: nuclear magnetic resonance
PRM: parallel reaction monitoring
STD: standard
SPE: solid-phase extraction

## References

[ref1] World Cancer Research Fund/American Institute for Cancer Research. Food, Nutrition, Physical Activity and the Prevention of Cancer: A Global Perspective; AICR: Washington DC, 2007.

[ref2] Rappaport S. M. Genetic Factors Are Not the Major Causes of Chronic Diseases. PLoS One 2016, 11, e0154387 10.1371/journal.pone.0154387. 27105432 PMC4841510

[ref3] Wishart D. Metabolomics and the Multi-Omics View of Cancer. Metabolites 2022, 12, 154 10.3390/metabo12020154. 35208228 PMC8880085

[ref4] Higginson J. Present and Future Developments in Environmental Carcinogenesis. In Epidemiology; Birch J. M., Ed.; Pergamon, 1979; pp 203–213 10.1016/B978-0-08-024386-3.50032-6.

[ref5] Maugh T. H. Cancer and Environment: Higginson Speaks Out. Science 1979, 205, 1363–1364. 10.1126/science.472753. 472753

[ref6] Wild C. P. Complementing the Genome with an “Exposome”: The Outstanding Challenge of Environmental Exposure Measurement in Molecular Epidemiology. Cancer Epidemiol., Biomarkers Prev. 2005, 14, 1847–1850. 10.1158/1055-9965.EPI-05-0456. 16103423

[ref7] Wild C. P. The Exposome: From Concept to Utility. Int. J. Epidemiol. 2012, 41, 24–32. 10.1093/ije/dyr236. 22296988

[ref8] González-Domínguez R.; Jáuregui O.; Queipo-Ortuño M. I.; Andrés-Lacueva C. Characterization of the Human Exposome by a Comprehensive and Quantitative Large-Scale Multianalyte Metabolomics Platform. Anal. Chem. 2020, 92, 13767–13775. 10.1021/acs.analchem.0c02008. 32966057

[ref9] Vrijheid M.; Fossati S.; Maitre L.; Márquez S.; Roumeliotaki T.; Agier L.; Andrusaityte S.; Cadiou S.; Casas M.; de Castro M.; Dedele A.; Donaire-Gonzalez D.; Grazuleviciene R.; Haug L. S.; McEachan R.; Meltzer H. M.; Papadopouplou E.; Robinson O.; Sakhi A. K.; Siroux V.; Sunyer J.; Schwarze P. E.; Tamayo-Uria I.; Urquiza J.; Vafeiadi M.; Valentin A.; Warembourg C.; Wright J.; Nieuwenhuijsen M. J.; Thomsen C.; Basagaña X.; Slama R.; Chatzi L. Early-Life Environmental Exposures and Childhood Obesity: An Exposome-Wide Approach. Environ. Health Perspect. 2020, 128, 067009 10.1289/EHP5975. 32579081 PMC7313401

[ref10] Zhang P.; Carlsten C.; Chaleckis R.; Hanhineva K.; Huang M.; Isobe T.; Koistinen V. M.; Meister I.; Papazian S.; Sdougkou K.; Xie H.; Martin J. W.; Rappaport S. M.; Tsugawa H.; Walker D. I.; Woodruff T. J.; Wright R. O.; Wheelock C. E. Defining the Scope of Exposome Studies and Research Needs from a Multidisciplinary Perspective. Environ. Sci. Technol. Lett. 2021, 8, 839–852. 10.1021/acs.estlett.1c00648. 34660833 PMC8515788

[ref11] Xue J.; Lai Y.; Liu C.-W.; Ru H. Towards Mass Spectrometry-Based Chemical Exposome: Current Approaches, Challenges, and Future Directions. Toxics 2019, 7, 41 10.3390/toxics7030041. 31426576 PMC6789759

[ref12] Rappaport S. M.; Barupal D. K.; Wishart D.; Vineis P.; Scalbert A. The Blood Exposome and Its Role in Discovering Causes of Disease. Environ. Health Perspect. 2014, 122, 769–774. 10.1289/ehp.1308015. 24659601 PMC4123034

[ref13] Enoch S. J.; Ellison C. M.; Schultz T. W.; Cronin M. T. D. A Review of the Electrophilic Reaction Chemistry Involved in Covalent Protein Binding Relevant to Toxicity. Crit. Rev. Toxicol. 2011, 41, 783–802. 10.3109/10408444.2011.598141. 21809939

[ref14] Enoch S. J.; Cronin M. T. D. A Review of the Electrophilic Reaction Chemistry Involved in Covalent DNA Binding. Crit. Rev. Toxicol. 2010, 40, 728–748. 10.3109/10408444.2010.494175. 20722585

[ref15] Tretyakova N.; Villalta P. W.; Kotapati S. Mass Spectrometry of Structurally Modified DNA. Chem. Rev. 2013, 113, 2395–2436. 10.1021/cr300391r. 23441727 PMC3700556

[ref16] Törnqvist M.; Fred C.; Haglund J.; Helleberg H.; Paulsson B.; Rydberg P. Protein Adducts: Quantitative and Qualitative Aspects of Their Formation, Analysis and Applications. J. Chromatogr. B 2002, 778, 279–308. 10.1016/S1570-0232(02)00172-1. 12376136

[ref17] Rubino F. M.; Pitton M.; Di Fabio D.; Colombi A. Toward an “omic” physiopathology of reactive chemicals: Thirty years of mass spectrometric study of the protein adducts with endogenous and xenobiotic compounds. Mass Spectrom. Rev. 2009, 28, 725–784. 10.1002/mas.20207. 19127566

[ref18] Sabbioni G.; Turesky R. J. Biomonitoring Human Albumin Adducts: The Past, the Present, and the Future. Chem. Res. Toxicol. 2017, 30, 332–366. 10.1021/acs.chemrestox.6b00366. 27989119 PMC5241710

[ref19] Ohnishi I.; Iwashita Y.; Matsushita Y.; Ohtsuka S.; Yamashita T.; Inaba K.; Fukazawa A.; Ochiai H.; Matsumoto K.; Kurono N.; Matsushima Y.; Mori H.; Suzuki S.; Suzuki S.; Tanioka F.; Sugimura H. Mass Spectrometric Profiling of DNA Adducts in the Human Stomach Associated with Damage from Environmental Factors. Genes Environ. 2021, 43, 12 10.1186/s41021-021-00186-2. 33836837 PMC8034090

[ref20] Guidolin V.; Carlson E. S.; Carrà A.; Villalta P. W.; Maertens L. A.; Hecht S. S.; Balbo S. Identification of New Markers of Alcohol-Derived DNA Damage in Humans. Biomolecules 2021, 11, 366 10.3390/biom11030366. 33673538 PMC7997542

[ref21] Totsuka Y.; Watanabe M.; Lin Y. New Horizons of DNA Adductome for Exploring Environmental Causes of Cancer. Cancer Sci. 2021, 112, 7–15. 10.1111/cas.14666. 32978845 PMC7780056

[ref22] Gorokhova E.; Martella G.; Motwani N. H.; Tretyakova N. Y.; Sundelin B.; Motwani H. V. DNA Epigenetic Marks Are Linked to Embryo Aberrations in Amphipods. Sci. Rep. 2020, 10, 655 10.1038/s41598-020-57465-1. 31959811 PMC6971077

[ref23] Balbo S.; Turesky R. J.; Villalta P. W. DNA Adductomics. Chem. Res. Toxicol. 2014, 27, 356–366. 10.1021/tx4004352. 24437709 PMC3997222

[ref24] Sousa P. F. M.; Martella G.; Åberg K. M.; Esfahani B.; Motwani H. V. NLossFinder—A Graphical User Interface Program for the Nontargeted Detection of DNA Adducts. Toxics 2021, 9, 78 10.3390/toxics9040078. 33916914 PMC8067598

[ref25] Walmsley S. J.; Guo J.; Murugan P.; Weight C. J.; Wang J.; Villalta P. W.; Turesky R. J. Comprehensive Analysis of DNA Adducts Using Data-Independent WSIM/MS2 Acquisition and WSIM-City. Anal. Chem. 2021, 93, 6491–6500. 10.1021/acs.analchem.1c00362. 33844920 PMC8675643

[ref26] Carlsson H.; Rappaport S. M.; Törnqvist M. Protein Adductomics: Methodologies for Untargeted Screening of Adducts to Serum Albumin and Hemoglobin in Human Blood Samples. High-Throughput 2019, 8, 6 10.3390/ht8010006. 30857166 PMC6473736

[ref27] Rappaport S. M.; Li H.; Grigoryan H.; Funk W. E.; Williams E. R. Adductomics: Characterizing Exposures to Reactive Electrophiles. Toxicol. Lett. 2012, 213, 83–90. 10.1016/j.toxlet.2011.04.002. 21501670 PMC4758449

[ref28] Grigoryan H.; Edmands W.; Lu S. S.; Yano Y.; Regazzoni L.; Iavarone A. T.; Williams E. R.; Rappaport S. M. Adductomics Pipeline for Untargeted Analysis of Modifications to Cys34 of Human Serum Albumin. Anal. Chem. 2016, 88, 10504–10512. 10.1021/acs.analchem.6b02553. 27684351 PMC5555296

[ref29] Preston G. W.; Dagnino S.; Ponzi E.; Sozeri O.; van Veldhoven K.; Barratt B.; Liu S.; Grigoryan H.; Lu S. S.; Rappaport S. M.; Chung K. F.; Cullinan P.; Sinharay R.; Kelly F. J.; Chadeau-Hyam M.; Vineis P.; Phillips D. H. Relationships between Airborne Pollutants, Serum Albumin Adducts and Short-Term Health Outcomes in an Experimental Crossover Study. Chemosphere 2020, 239, 124667 10.1016/j.chemosphere.2019.124667. 31499299

[ref30] Grigoryan H.; Edmands W. M. B.; Lan Q.; Carlsson H.; Vermeulen R.; Zhang L.; Yin S.-N.; Li G.-L.; Smith M. T.; Rothman N.; Rappaport S. M. Adductomic Signatures of Benzene Exposure Provide Insights into Cancer Induction. Carcinogenesis 2018, 39, 661–668. 10.1093/carcin/bgy042. 29538615 PMC5932554

[ref31] Dagnino S.; Bodinier B.; Grigoryan H.; Rappaport S. M.; Karimi M.; Guida F.; Polidoro S.; Edmands Wi. B.; Naccarati A.; Fiorito G.; Sacerdote C.; Krogh V.; Vermeulen R.; Vineis P.; Chadeau-Hyam M. Agnostic Cys34-Albumin Adductomics and DNA Methylation: Implication of N-Acetylcysteine in Lung Carcinogenesis Years before Diagnosis. Int. J. Cancer 2020, 146, 3294–3303. 10.1002/ijc.32680. 31513294

[ref32] Yano Y.; Schiffman C.; Grigoryan H.; Hayes J.; Edmands W.; Petrick L.; Whitehead T.; Metayer C.; Dudoit S.; Rappaport S. Untargeted Adductomics of Newborn Dried Blood Spots Identifies Modifications to Human Serum Albumin Associated with Childhood Leukemia. Leuk. Res. 2020, 88, 106268 10.1016/j.leukres.2019.106268. 31760269 PMC6937378

[ref33] Rydberg P.; von Stedingk H.; Magnér J.; Björklund J. LC/MS/MS Analysis of N-Terminal Protein Adducts with Improved Sensitivity: A Comparison of Selected Edman Isothiocyanate Reagents. Int. J. Anal. Chem. 2009, 2009, e153472 10.1155/2009/153472. PMC2809355 20107558

[ref34] von Stedingk H.; Rydberg P.; Törnqvist M. A New Modified Edman Procedure for Analysis of N-Terminal Valine Adducts in Hemoglobin by LC–MS/MS. J. Chromatogr. B 2010, 878, 2483–2490. 10.1016/j.jchromb.2010.03.034. 20399714

[ref35] von Stedingk H.; Vikström A. C.; Rydberg P.; Pedersen M.; Nielsen J. K. S.; Segerbäck D.; Knudsen L. E.; Törnqvist M. Analysis of Hemoglobin Adducts from Acrylamide, Glycidamide, and Ethylene Oxide in Paired Mother/Cord Blood Samples from Denmark. Chem. Res. Toxicol. 2011, 24, 1957–1965. 10.1021/tx200284u. 21882862

[ref36] Carlsson H.; von Stedingk H.; Nilsson U.; Törnqvist M. LC–MS/MS Screening Strategy for Unknown Adducts to N-Terminal Valine in Hemoglobin Applied to Smokers and Nonsmokers. Chem. Res. Toxicol. 2014, 27, 2062–2070. 10.1021/tx5002749. 25350717

[ref37] Carlsson H.; Aasa J.; Kotova N.; Vare D.; Sousa P. F. M.; Rydberg P.; Abramsson-Zetterberg L.; Törnqvist M. Adductomic Screening of Hemoglobin Adducts and Monitoring of Micronuclei in School-Age Children. Chem. Res. Toxicol. 2017, 30, 1157–1167. 10.1021/acs.chemrestox.6b00463. 28398741

[ref38] Carlsson H.; Törnqvist M. Strategy for Identifying Unknown Hemoglobin Adducts Using Adductome LC-MS/MS Data: Identification of Adducts Corresponding to Acrylic Acid, Glyoxal, Methylglyoxal, and 1-Octen-3-One. Food Chem. Toxicol. 2016, 92, 94–103. 10.1016/j.fct.2016.03.028. 27046699

[ref39] Pedersen M.; Vryonidis E.; Joensen A.; Törnqvist M. Hemoglobin Adducts of Acrylamide in Human Blood – What Has Been Done and What Is Next?. Food Chem. Toxicol. 2022, 161, 112799 10.1016/j.fct.2021.112799. 34995709

[ref40] Aasa J.; Abramsson-Zetterberg L.; Carlsson H.; Törnqvist M. The Genotoxic Potency of Glycidol Established from Micronucleus Frequency and Hemoglobin Adduct Levels in Mice. Food Chem. Toxicol. 2017, 100, 168–174. 10.1016/j.fct.2016.12.022. 28012894

[ref41] Aasa J.; Vryonidis E.; Abramsson-Zetterberg L.; Törnqvist M. Internal Doses of Glycidol in Children and Estimation of Associated Cancer Risk. Toxics 2019, 7, 7 10.3390/toxics7010007. 30717263 PMC6468878

[ref42] Glynn A.; Kotova N.; Dahlgren E.; Lindh C.; Jakobsson K.; Gyllenhammar I.; Lignell S.; Nälsén C. Determinants of Serum Concentrations of Perfluoroalkyl Acids (PFAAs) in School Children and the Contribution of Low-Level PFAA-Contaminated Drinking Water. Environ. Sci. Process. Impacts 2020, 22, 930–944. 10.1039/C9EM00497A. 32040098

[ref43] Nälsén C.; Becker W.; Pearson M.; Ridefelt P.; Lindroos A. K.; Kotova N.; Mattisson I. Vitamin D Status in Children and Adults in Sweden: Dietary Intake and 25-Hydroxyvitamin D Concentrations in Children Aged 10–12 Years and Adults Aged 18–80 Years. J. Nutr. Sci. 2020, 9, e47 10.1017/jns.2020.40. 33101664 PMC7550965

[ref44] Aasa J.; Granath F.; Törnqvist M. Cancer Risk Estimation of Glycidol Based on Rodent Carcinogenicity Studies, a Multiplicative Risk Model and in Vivo Dosimetry. Food Chem. Toxicol. 2019, 128, 54–60. 10.1016/j.fct.2019.03.037. 30914355

[ref45] Törnqvist M.; Paulsson B.; Vikström A. C.; Granath F. Approach for Cancer Risk Estimation of Acrylamide in Food on the Basis of Animal Cancer Tests and in Vivo Dosimetry. J. Agric. Food Chem. 2008, 56, 6004–6012. 10.1021/jf800490s. 18624431

[ref46] Rydberg P. Method for Analyzing N-Terminal Protein Adducts. EP1738177, 2009.

[ref47] Sabbioni G.; Berset J.-D.; Day B. W. Is It Realistic to Propose Determination of a Lifetime Internal Exposome?. Chem. Res. Toxicol. 2020, 33, 2010–2021. 10.1021/acs.chemrestox.0c00092. 32672951

[ref48] Calleman C. J. Hemoglobin as a Dose Monitor and Its Application to the Risk Estimation of Ethylene Oxide, Stockholm University, 1984.

[ref49] Rydberg P.; Lüning B.; Wachtmeister C. A.; Törnqvist M.; Lönnberg H.; Led J. J.; MIlanova R. K.; Nakata H.; Nasiri A.; Tsuda T. Synthesis and Characterization of N-Substituted Valines and Their Phenyl- and Pentafluorophenyl-Thiohydantoins. Acta Chem. Scand. 1993, 47, 813–817. 10.3891/acta.chem.scand.47-0813.

[ref50] Vikström A. C.; Abramsson-Zetterberg L.; Naruszewicz M.; Athanassiadis I.; Granath F. N.; Törnqvist MÅ. In Vivo Doses of Acrylamide and Glycidamide in Humans after Intake of Acrylamide-Rich Food. Toxicol. Sci. 2011, 119, 41–49. 10.1093/toxsci/kfq323. 20952504

[ref51] Bergmark E. Hemoglobin Adducts of Acrylamide and Acrylonitrile in Laboratory Workers, Smokers and Nonsmokers. Chem. Res. Toxicol. 1997, 10, 78–84. 10.1021/tx960113p. 9074806

[ref52] Scherer G.; Engl J.; Urban M.; Gilch G.; Janket D.; Riedel K. Relationship between Machine-Derived Smoke Yields and Biomarkers in Cigarette Smokers in Germany. Regul. Toxicol. Pharmacol. 2007, 47, 171–183. 10.1016/j.yrtph.2006.09.001. 17034917

[ref53] Hindsø Landin H.; Grummt T.; Laurent C.; Tates A. Monitoring of Occupational Exposure to Epichlorohydrin by Genetic Effects and Hemoglobin Adducts. Mutat. Res., Fundam. Mol. Mech. Mutagen. 1997, 381, 217–226. 10.1016/S0027-5107(97)00171-1. 9434878

[ref54] Cheng W.-w.; Liu G.; Wang L.; Liu Z. Glycidyl Fatty Acid Esters in Refined Edible Oils: A Review on Formation, Occurrence, Analysis, and Elimination Methods. Compr. Rev. Food Sci. Food Saf. 2017, 16, 263–281. 10.1111/1541-4337.12251. 33371535

[ref55] Risks for Human Health Related to the Presence of 3- and 2-monochloropropanediol (MCPD), and Their Fatty Acid Esters, and Glycidyl Fatty Acid Esters in Food. EFSA J. 2016, 14, e04426 10.2903/j.efsa.2016.4426.

[ref56] Tareke E.; Rydberg P.; Karlsson P.; Eriksson S.; Törnqvist M. Analysis of Acrylamide, a Carcinogen Formed in Heated Foodstuffs. J. Agric. Food Chem. 2002, 50, 4998–5006. 10.1021/jf020302f. 12166997

[ref57] Scientific Opinion on Acrylamide in Food. EFSA J. 2015, 13, 4104 10.2903/j.efsa.2015.4104.

[ref58] Timmermann C. A. G.; Mølck S. S.; Kadawathagedara M.; Bjerregaard A. A.; Törnqvist M.; Brantsæter A. L.; Pedersen M. A Review of Dietary Intake of Acrylamide in Humans. Toxics 2021, 9, 155 10.3390/toxics9070155. 34209352 PMC8309717

[ref59] Charoenprasert S.; Zweigenbaum J. A.; Zhang G.; Mitchell A. E. The Influence of pH and Sodium Hydroxide Exposure Time on Glucosamine and Acrylamide Levels in California-Style Black Ripe Olives. J. Food Sci. 2017, 82, 1574–1581. 10.1111/1750-3841.13748. 28556254

[ref60] Schumacher J. N.; Green C. R.; Best F. W.; Newell M. P. Smoke Composition. An Extensive Investigation of the Water-Soluble Portion of Cigarette Smoke. J. Agric. Food Chem. 1977, 25, 310–320. 10.1021/jf60210a003. 838966

[ref61] Smith C. J.; Perfetti T. A.; Rumple M. A.; Rodgman A.; Doolittle D. J. “IARC Group 2A Carcinogens” Reported in Cigarette Mainstream Smoke. Food Chem. Toxicol. 2000, 38, 371–383. 10.1016/S0278-6915(99)00156-8. 10722891

[ref62] Esposito F.; Squillante J.; Nolasco A.; Montuori P.; Macrì P. G.; Cirillo T. Acrylamide Levels in Smoke from Conventional Cigarettes and Heated Tobacco Products and Exposure Assessment in Habitual Smokers. Environ. Res. 2022, 208, 112659 10.1016/j.envres.2021.112659. 34990604

[ref63] IARC. Agents Classified by the IARC Monographs, Volumes 1–130 – IARC Monographs on the Identification of Carcinogenic Hazards to Humans. IARC Monographs on the Evaluation of Risk to Humans. https://monographs.iarc.who.int/agents-classified-by-the-iarc/ (accessed Feb 15, 2022).

[ref64] Wandel U.; Machado S. S.; Jongejan J. A.; Duine J. A. Enzymes Involved in the Glycidaldehyde (2,3-Epoxy-Propanal) Oxidation Step in the Kinetic Resolution of Racemic Glycidol (2,3-Epoxy-1-Propanol) by Acetobacter Pasteurianus. Enzyme Microb. Technol. 2001, 28, 233–239. 10.1016/S0141-0229(00)00321-5. 11166817

[ref65] Vikström A. C.; Warholm M.; Paulsson B.; Axmon A.; Wirfält E.; Törnqvist M. Hemoglobin Adducts as a Measure of Variations in Exposure to Acrylamide in Food and Comparison to Questionnaire Data. Food Chem. Toxicol. 2012, 50, 2531–2539. 10.1016/j.fct.2012.04.004. 22525869

[ref66] Pellè L.; Carlsson H.; Cipollini M.; Bonotti A.; Foddis R.; Cristaudo A.; Romei C.; Elisei R.; Gemignani F.; Törnqvist M.; Landi S. The Polymorphism Rs2480258 within CYP2E1 Is Associated with Different Rates of Acrylamide Metabolism in Vivo in Humans. Arch. Toxicol. 2018, 92, 2137–2140. 10.1007/s00204-018-2211-2. 29748789

[ref67] Carlsson H.; Motwani H. V.; Osterman Golkar S.; Törnqvist M. Characterization of a Hemoglobin Adduct from Ethyl Vinyl Ketone Detected in Human Blood Samples. Chem. Res. Toxicol. 2015, 28, 2120–2129. 10.1021/acs.chemrestox.5b00287. 26447499

[ref68] Wishart D. S.; Guo A.; Oler E.; Wang F.; Anjum A.; Peters H.; Dizon R.; Sayeeda Z.; Tian S.; Lee B. L.; Berjanskii M.; Mah R.; Yamamoto M.; Jovel J.; Torres-Calzada C.; Hiebert-Giesbrecht M.; Lui V. W.; Varshavi D.; Varshavi D.; Allen D.; Arndt D.; Khetarpal N.; Sivakumaran A.; Harford K.; Sanford S.; Yee K.; Cao X.; Budinski Z.; Liigand J.; Zhang L.; Zheng J.; Mandal R.; Karu N.; Dambrova M.; Schiöth H. B.; Greiner R.; Gautam V. HMDB 5.0: The Human Metabolome Database for 2022. Nucleic Acids Res. 2022, 50, D622–D631. 10.1093/nar/gkab1062. 34986597 PMC8728138

[ref69] Degner A.; Carlsson H.; Karlsson I.; Eriksson J.; Pujari S. S.; Tretyakova N. Y.; Törnqvist M. Discovery of Novel N-(4-Hydroxybenzyl)Valine Hemoglobin Adducts in Human Blood. Chem. Res. Toxicol. 2018, 31, 1305–1314. 10.1021/acs.chemrestox.8b00173. 30375232 PMC6424601

[ref70] Törnqvist M. Formation of Reactive Species That Lead to Hemoglobin Adducts during Strong of Blood Samples. Carcinogenesis 1990, 11, 51–54. 10.1093/carcin/11.1.51. 2295127

[ref71] Kautiainen A.; Osterman-Golkar S.; Ehrenberg L.; Christophersen C.; Krogsgaard-Larsen P.; Ryhage R.; Isaksson R. Misincorporation of Alkylated Amino Acids into Hemoglobin -- a Possible Source of Background Alkylations. Acta Chem. Scand. 1986, 40b, 453–456. 10.3891/acta.chem.scand.40b-0453. 3766015

[ref72] Kautiainen A.; Fred C.; Rydberg P.; Törnqvist M. A Liquid Chromatography Tandem Mass Spectrometric Method for in Vivo Dose Monitoring of Diepoxybutane, a Metabolite of Butadiene. Rapid Commun. Mass Spectrom. 2000, 14, 1848–1853. 10.1002/1097-0231(20001015)14:19%3C1848::AID-RCM106%3E3.0.CO;2-%23. 11006595

[ref73] Boysen G.; Shimoni A.; Danylesko I.; Varda-Bloom N.; Nagler A. A Simplified Method for Detection of N-Terminal Valine Adducts in Patients Receiving Treosulfan. Rapid Commun. Mass Spectrom. 2019, 33, 1635–1642. 10.1002/rcm.8509. 31240802 PMC6817381

[ref74] Chung M. K.; Riby J.; Li H.; Iavarone A. T.; Williams E. R.; Zheng Y.; Rappaport S. M. A Sandwich Enzyme-Linked Immunosorbent Assay for Adducts of Polycyclic Aromatic Hydrocarbons with Human Serum Albumin. Anal. Biochem. 2010, 400, 123–129. 10.1016/j.ab.2010.01.018. 20083082 PMC2842209

[ref75] Motwani H. V.; Westberg E.; Törnqvist M. Interaction of Benzo [a] Pyrene Diol Epoxide Isomers with Human Serum Albumin: Site Specific Characterisation of Adducts and Associated Kinetics. Sci. Rep. 2016, 6, 36243 10.1038/srep36243. 27805056 PMC5090251

[ref76] Sabbioni G. Hemoglobin Adducts and Urinary Metabolites of Arylamines and Nitroarenes. Chem. Res. Toxicol. 2017, 30, 1733–1766. 10.1021/acs.chemrestox.7b00111. 28933159

[ref77] Segerbäck D. Reaction Products in Hemoglobin and DNA after in Vitro Treatment with Ethylene Oxide and N-(2-Hydroxyethyl)-N-Nitrosourea. Carcinogenesis 1990, 11, 307–312. 10.1093/carcin/11.2.307. 2302758

[ref78] Rajczewski A. T.; Ndreu L.; Pujari S. S.; Griffin T. J.; Törnqvist M. Å.; Karlsson I.; Tretyakova N. Y. Novel 4-Hydroxybenzyl Adducts in Human Hemoglobin: Structures and Mechanisms of Formation. Chem. Res. Toxicol. 2021, 34, 1769–1781. 10.1021/acs.chemrestox.1c00111. 34110810 PMC10159211

[ref79] Ndreu L.; Erber L. N.; Törnqvist M.; Tretyakova N. Y.; Karlsson I. Characterizing Adduct Formation of Electrophilic Skin Allergens with Human Serum Albumin and Hemoglobin. Chem. Res. Toxicol. 2020, 33, 2623–2636. 10.1021/acs.chemrestox.0c00271. 32875789 PMC7582624

[ref80] Antunes A. M. M.; Godinho A. L. A.; Martins I. L.; Oliveira M. C.; Gomes R. A.; Coelho A. V.; Beland F. A.; Marques M. M. Protein Adducts As Prospective Biomarkers of Nevirapine Toxicity. Chem. Res. Toxicol. 2010, 23, 1714–1725. 10.1021/tx100186t. 20809596 PMC2981636

[ref81] Grigoryan H.; Imani P.; Dudoit S.; Rappaport S. M. Extending the HSA-Cys34-Adductomics Pipeline to Modifications at Lys525. Chem. Res. Toxicol. 2021, 34, 2549–2557. 10.1021/acs.chemrestox.1c00311. 34788011

[ref82] Kojima K.; Lee S. H.; Oe T. An LC/ESI-SRM/MS Method to Screen Chemically Modified Hemoglobin: Simultaneous Analysis for Oxidized, Nitrated, Lipidated, and Glycated Sites. Anal. Bioanal. Chem. 2016, 408, 5379–5392. 10.1007/s00216-016-9635-4. 27236314

