# Supplementary material for: Pathways to Identify Electrophiles In Vivo Using Hemoglobin Adducts: Hydroxypropanoic Acid Valine Adduct and Its Possible Precursors
Source: Chem Res Toxicol. 2022 Nov 17;35(12):2227–40. doi: 10.1021/acs.chemrestox.2c00208 (PMC9768813; doi:10.1021/acs.chemrestox.2c00208)

Supporting Information

Pathways to identify electrophiles *in vivo* using hemoglobin adducts: Hydroxypropanoic acid valine adduct and its possible precursors

Efstathios Vryonidis[†], Isabella Karlsson[†], Jenny Aasa[‡], Henrik Carlsson[§], Hitesh V. Motwani[†], Marie Pedersen[¤], Johan Eriksson[†] and Margareta Å. Törnqvist[†*]

[†]Department of Environmental Science, Stockholm University, SE-106 91 Stockholm, Sweden

[‡]Department of Risk and Benefit Assessment, Swedish Food Agency, SE-751 26 Uppsala, Sweden

[§]Department of Medical Sciences, Clinical Chemistry, Uppsala University, SE-751 85 Uppsala, Sweden

[¤]Department of Public Health, University of Copenhagen, DK-1353 Copenhagen, Denmark

Table of Contents

S1. NMR spectra of the synthesized HPA-Val-FTH S2
S2. NMR analysis of the synthesized reference H-Val-FTH S3
S3. NMR analysis of the synthesized reference AA-Val-FTH S5
S4. NMR analysis of the synthesized reference ACA-Val-FTH S7
S5. Comparison of the NMR spectra of HPA-Val-FTH and the reference FTHs S9
S6. Illustration of synthetic pathways to HPA-Val S10
S7. Experiment in blood to estimate the rate constant for HPA-Val adduct formation from GLA ... S11
S8. HPA-Val adduct levels in rodents dosed with glycidol or acrylamide S12

S1. NMR spectra of the synthesized HPA-Val-FTH

a. ¹H NMR spectra

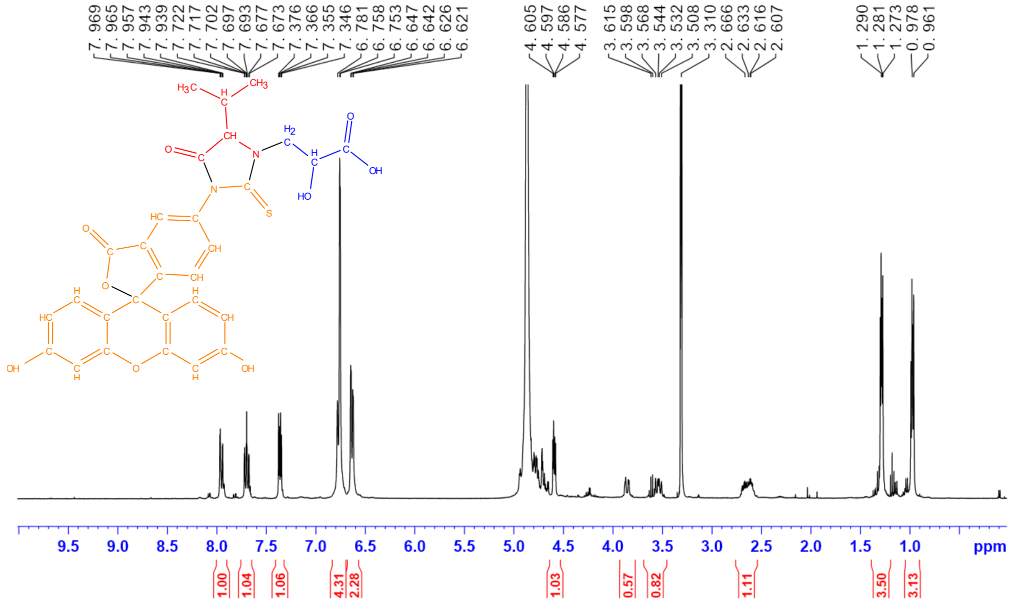

b. ¹³C NMR spectra

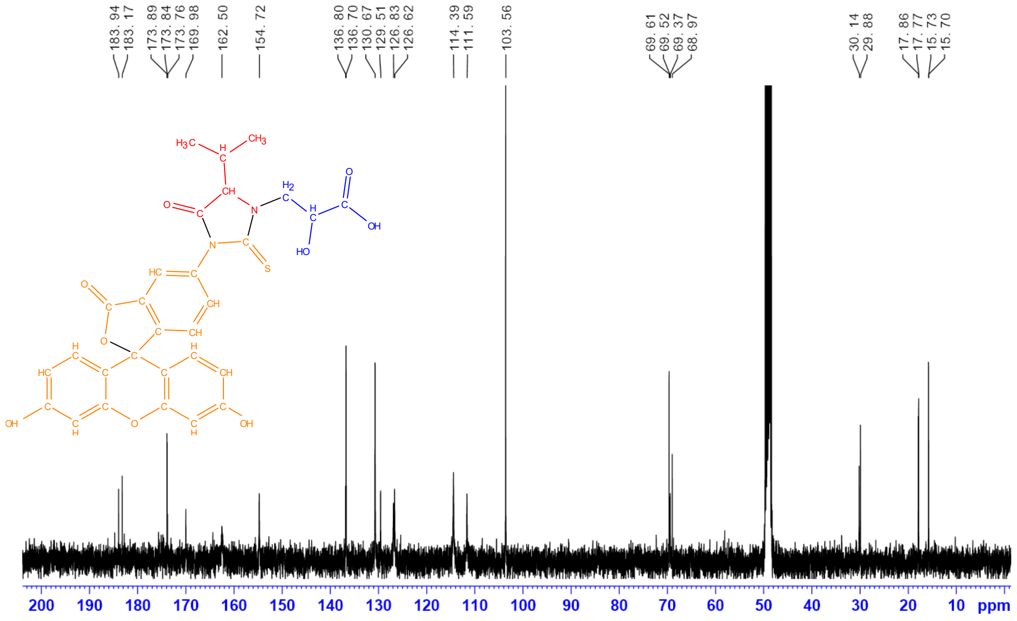

S2. NMR analysis of the synthesized reference H-Val-FTH

a. ^{1}H NMR spectra

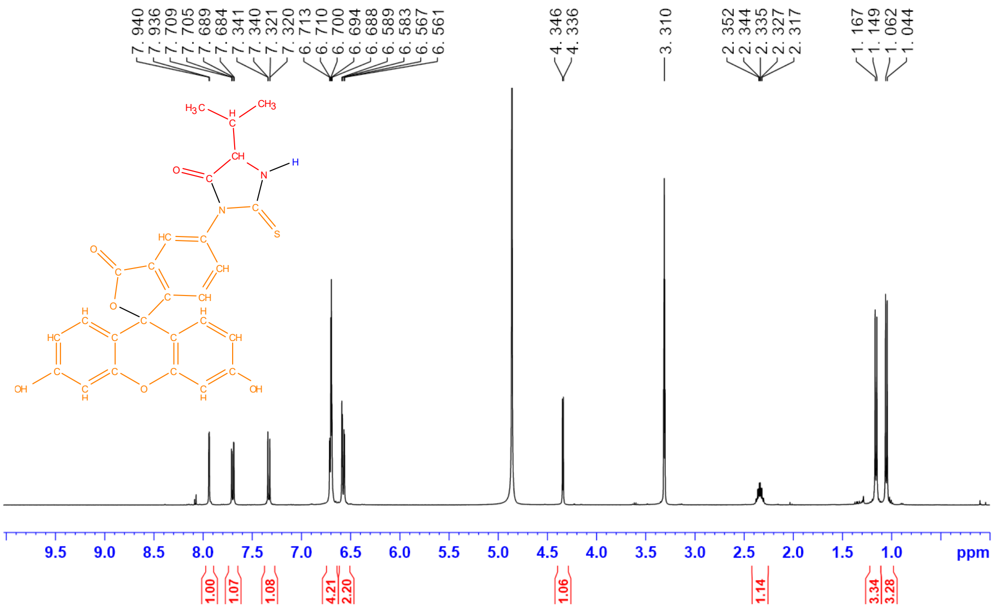

b. ^{13}C NMR spectra

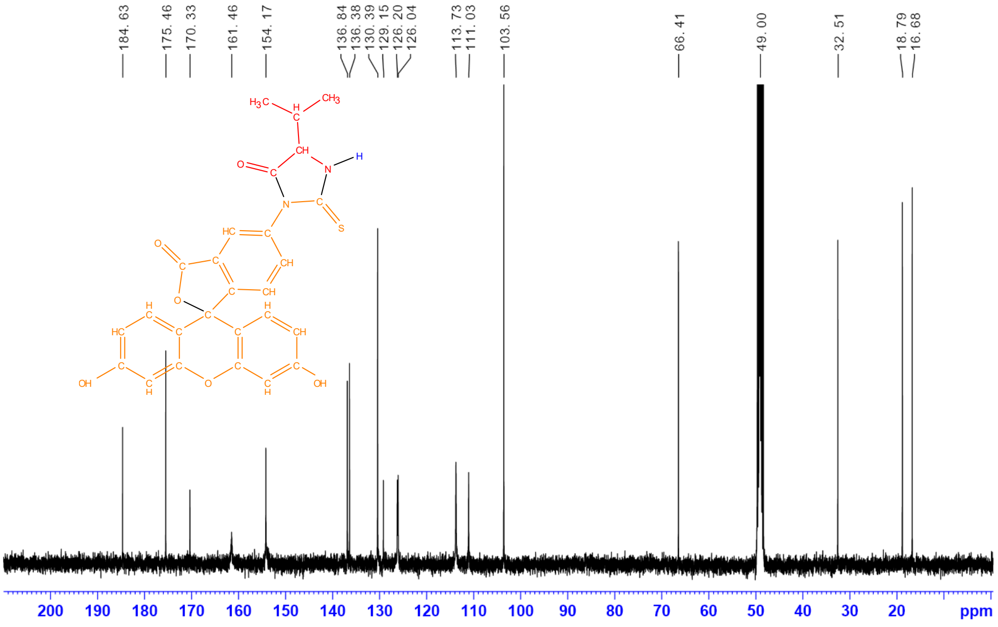

c. NMR characterization of H-Val-FTH

^{1}H NMR (400 MHz, CD3OD): δ 7.94 (dd, J = 2.0, 0.6 Hz, 1H), 7.70 (dd, J = 8.2, 1.9 Hz, 1H), 7.33 (dd, J = 8.1, 0.7 Hz, 1H), 6.69 (dd, J = 8.6, 1.5 Hz, 4H), 6.57 (dd, J = 8.7, 2.4 Hz, 2H), 4.34 (d, J = 3.8 Hz, 1H), 2.34 (pd, J = 6.9, 3.8 Hz, 1H), 1.16 (d, J = 7.0 Hz, 3H), 1.05 (d, J = 6.9 Hz, 3H).

^{13}C NMR (100 MHz, CD3OD): δ 184.63, 175.46, 170.33, 161.46, 154.17, 136.84, 136.38, 130.39, 129.16, 126.20, 126.04, 113.74, 111.03, 103.56, 66.42, 32.51, 18.79, 16.68.

S3. NMR analysis of the synthesized reference AA-Val-FTH

a. ¹H NMR spectra

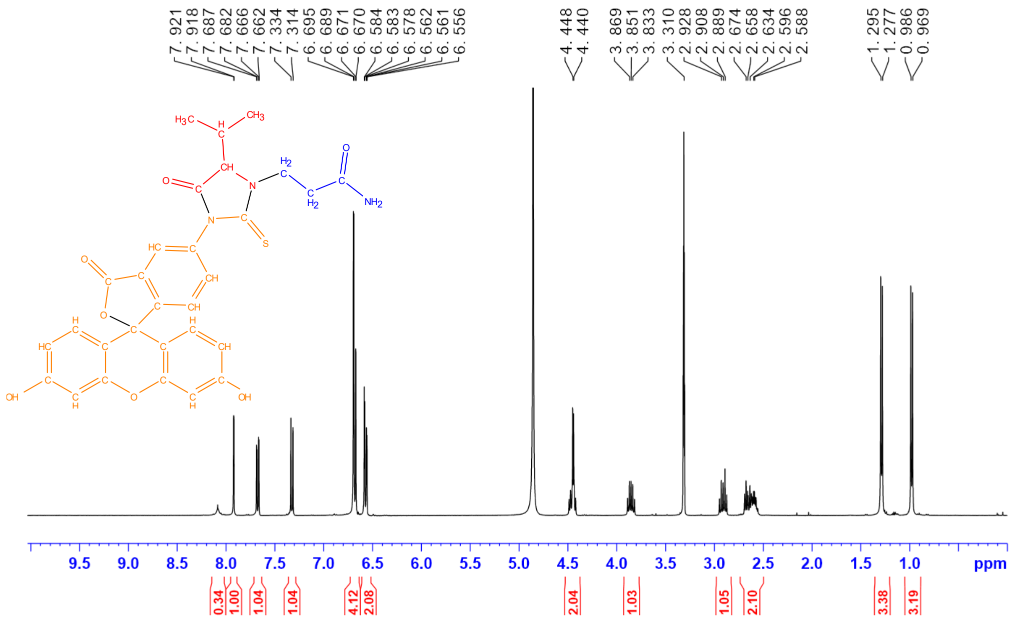

b. ¹³C NMR spectra

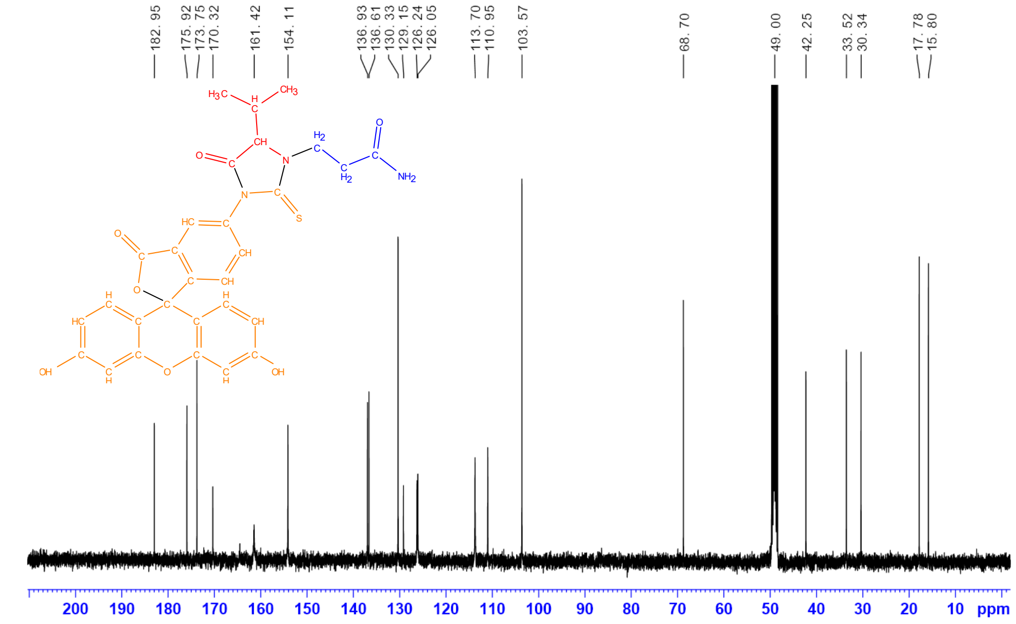

c. NMR characterization of AA-Val-FTH

^{1}H NMR (400 MHz, CD3OD): δ 7.92 (d, J = 1.8 Hz, 1H), 7.67 (dd, J = 8.1, 1.9 Hz, 1H), 7.32 (d, J = 8.1 Hz, 1H), 6.68 (dd, J = 8.6, 1.5 Hz, 4H), 6.57 (dd, J = 8.7, 2.4 Hz, 2H), 4.50-4.40 (m, 2H), 3.85 (dt, J = 14.3, 7.2 Hz, 1H), 2.91 (dt, J = 14.9, 7.3 Hz, 1H), 2.71 – 2.53 (m, 2H), 1.29 (d, J = 7.0 Hz, 3H), 0.98 (d, J = 6.8 Hz, 3H).

^{13}C NMR (100 MHz, CD3OD): δ 182.95, 175.92, 173.75, 170.32, 161.43, 154.11, 136.93, 136.61, 130.33, 129.15, 126.24, 126.05, 113.69, 110.95, 103.57, 68.70, 42.25, 33.52, 30.34, 17.78, 15.80.

S4. NMR analysis of the synthesized reference ACA-Val-FTH

a. ^{1}H NMR spectra

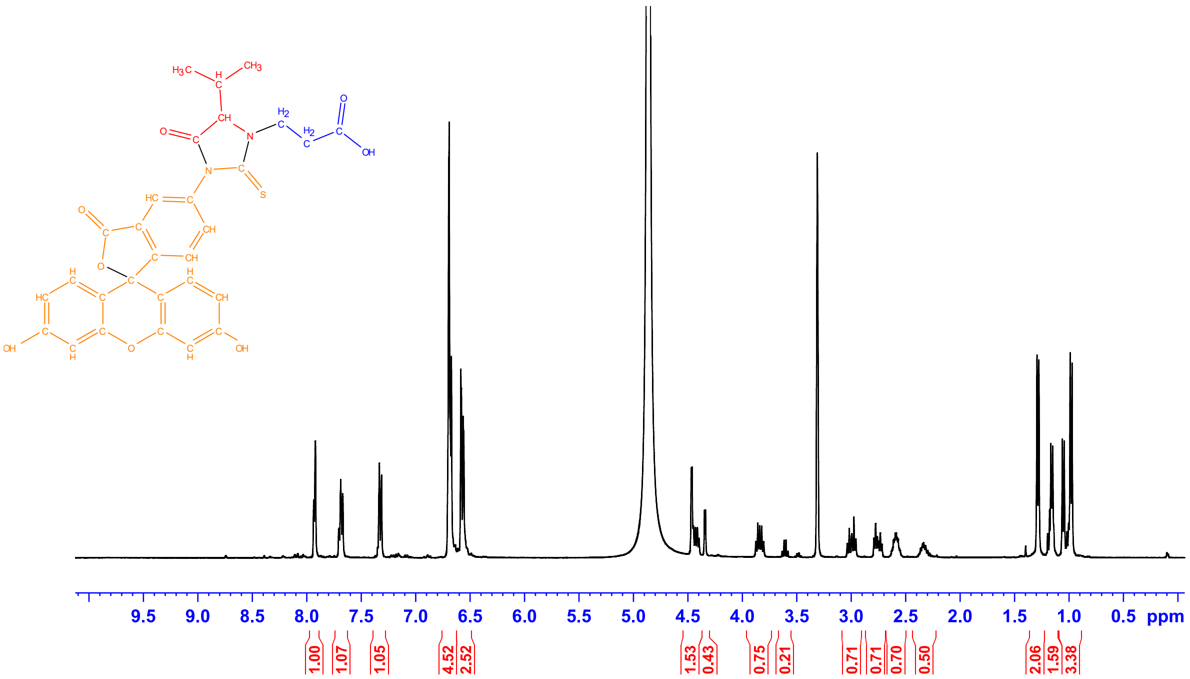

b. ^{13}C NMR spectra

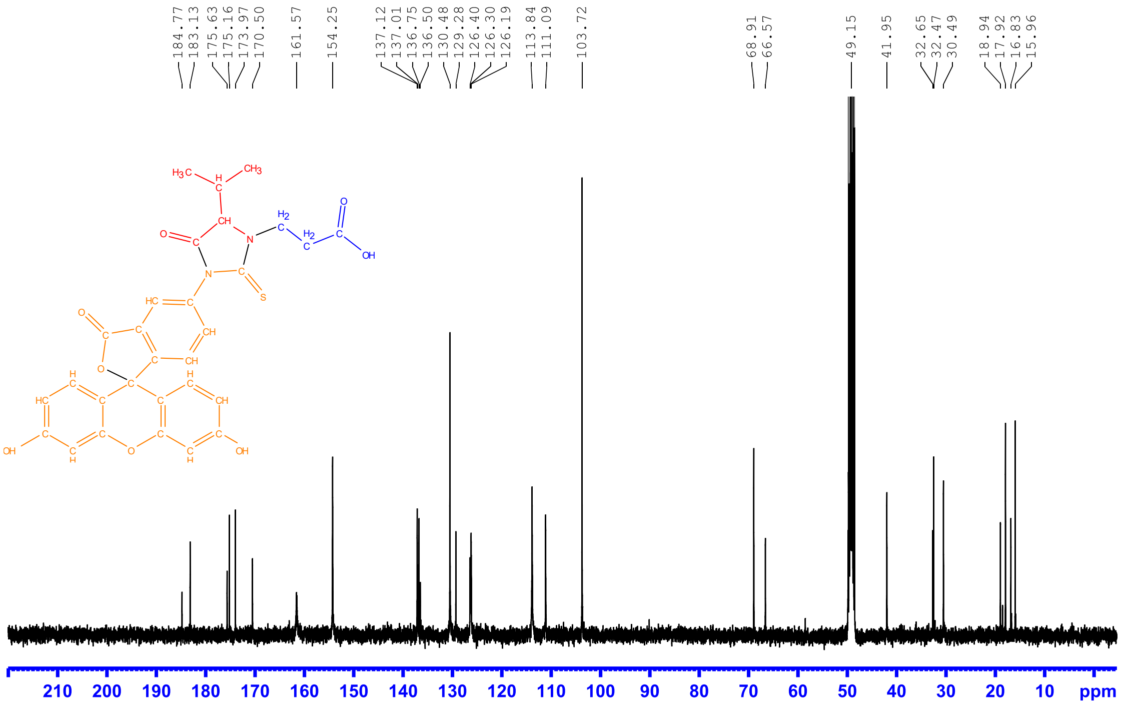

c. NMR characterization of ACA-Val-FTH

The product was not pure and contained H-Val-FTH. Peaks not listed originate from H-Val-FTH.

^{1}H NMR (400 MHz, CD3OD): δ 7.91 (d, J = 1.8 Hz, 1H), 7.68 (dd, J = 8.1, 1.9 Hz, 1H), 7.32 (d, J = 8.1 Hz, 1H), 6.68 (dd, J = 8.6, 1.5 Hz, 4H), 6.57 (dd, J = 8.7, 2.4 Hz, 2H), 4.47-4.41 (m, 2H), 3.84 (dt, J = 14.3, 7.2 Hz, 1H), 2.99 (dt, J = 14.9, 7.3 Hz, 1H), 2.79 – 2.72 (m, 1H), 2.61-2.57 (1H), 1.28 (d, J = 7.0 Hz, 3H), 0.99 (d, J = 6.8 Hz, 3H).

^{13}C NMR (100 MHz, CD3OD): δ 183.13, 175.16, 173.97, 170.50, 161.57, 154.26, 137.12, 136.75, 130.48, 129.28, 126.40, 126.19, 113.84, 111.09, 103.72, 68.92, 41.96, 32.47, 30.49, 17.92, 15.96.

S5. Comparison of the NMR spectra of HPA-Val-FTH and the reference FTHs

When comparing the ^{13}C NMR spectrum of H-Val-FTH with that of AA-Val-FTH they show good correspondence and include the expected three additional peaks in the ^{13}C NMR of AA-Val-FTH (see Figure SI 2b and SI 3b). The peak at δ 173.75, corresponds to the amide-C, whereas the two peaks at δ 42.25 and δ 33.52 corresponds to the two aliphatic carbons (α- and β-C to the carbonyl) of the acrylamide group. The ^{13}C NMR spectrum of HPA-Val-FTH (Figure S1b) is somewhat more complex due to the fact that HPA-Val-FTH is a mixture of isomers, which explains the double carbon signals seen for the isopropyl-thioxoimidazolidinone. As expected, signals corresponding to the carboxyl acid (δ 173-174) and the aliphatic carbons (δ 69-70) of the HPA-adduct can also be seen, which supports the identification.

In the ^{1}H NMR for AA-Val-FTH, the signals corresponding to the aromatic protons, as well as the protons of the isopropyl group can be seen at similar shifts as for the H-Val-FTH (see Figure S2 a and S3 a). In addition, four double triplets can be seen for each one of the aliphatic protons of the acrylamide group. One of these has a shift similar to the β-proton of the isopropyl group and one has a shift similar to that of the α-proton of the isopropyl group. That HPA-Val-FTH is a mixture of isomers, which makes the spectrum considerably more complex. However, peaks can be seen that correspond to the aliphatic protons of the HPA-Val adduct (δ 4.80-4.65, 3.88-3.83, and 3.58-3.49) (Figure S1 a); thus, confirming the structure of the HPA-Val adduct.

S6. Illustration of synthetic pathways to HPA-Val

Schematic illustration of three different synthetic pathways yielding the observed HPA-Val adduct:

(a) epoxide nucleophilic substitution reaction of ethyl oxirane-2-carboxylate with valine (requires hydrolysis of the ester bound in the second step);

(b) amine alkylation with alkyl halide reaction of isoserine with 2-bromoisovaleric acid, and;

(c) reductive amination reaction of 2-hydroxy-3-oxopropanoic acid with Val via Schiff base (which requires reduction in the second step).

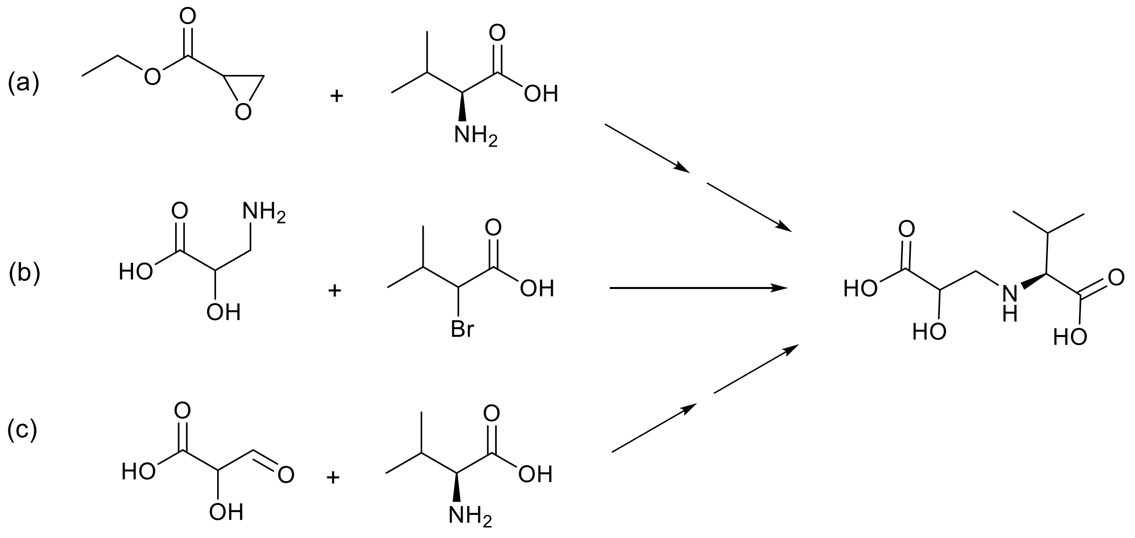

S7. Experiment in blood to estimate the rate constant for HPA-Val adduct formation from GLA

The rate constant for adduct formation toward N-terminal valines in Hb by GLA was estimated from *in vitro* studies under pseudo first-order reaction conditions in whole lysed blood with GLA. The normalized area of the HPA- Val-FTH analyte to $^{13}C_5$-substituted IS (GL IS) in A_x/A_{IS} is plotted against incubation time in h. The figure shows the data for five time points. Timepoints 0 and 0.17 h are omitted.

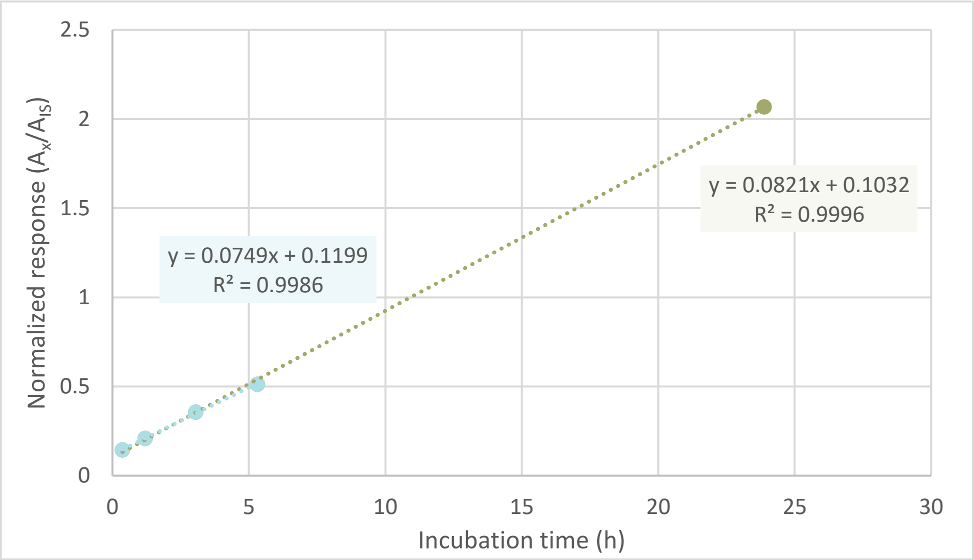

S8. HPA-Val adduct levels in rodents dosed with glycidol or acrylamide

Comparison of the HPA-Val adduct level increase relative to the control in rodents dosed with acrylamide (AA) and glycidol (GL). The exposure level is denoted LOW or HIGH, and control for no exposure, in mouse and rat, respectively.

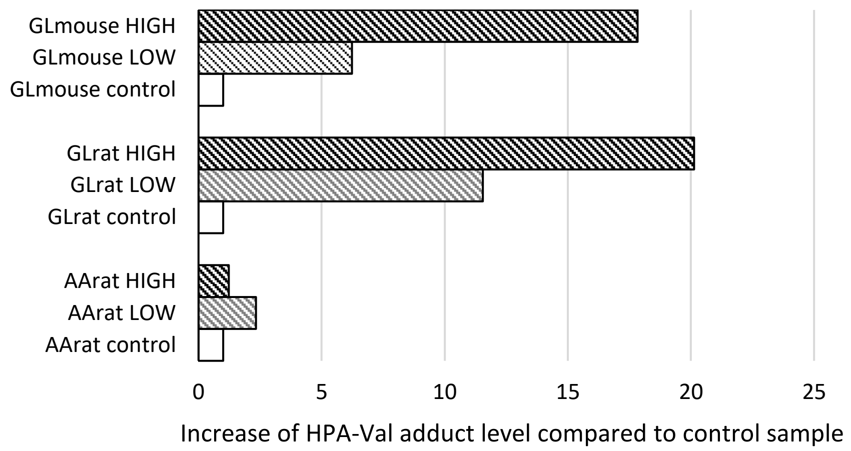

Supplement: Supplementary file 1 — tx2c00208_si_001.pdf [file tx2c00208_si_001.pdf]
